# Genome-Wide Macrosynteny among *Fusarium* Species in the *Gibberella fujikuroi* Complex Revealed by Amplified Fragment Length Polymorphisms

**DOI:** 10.1371/journal.pone.0114682

**Published:** 2014-12-08

**Authors:** Lieschen De Vos, Emma T. Steenkamp, Simon H. Martin, Quentin C. Santana, Gerda Fourie, Nicolaas A. van der Merwe, Michael J. Wingfield, Brenda D. Wingfield

**Affiliations:** 1 Department of Genetics, Forestry and Agricultural Biotechnology Institute (FABI), University of Pretoria, Private Bag X20, Pretoria, South Africa; 2 Department of Microbiology and Plant Pathology, Forestry and Agricultural Biotechnology Institute (FABI), University of Pretoria, Private Bag X20, Pretoria, South Africa; Ruhr-University Bochum, Germany

## Abstract

The *Gibberella fujikuroi* complex includes many *Fusarium* species that cause significant losses in yield and quality of agricultural and forestry crops. Due to their economic importance, whole-genome sequence information has rapidly become available for species including *Fusarium circinatum, Fusarium fujikuroi* and *Fusarium verticillioides*, each of which represent one of the three main clades known in this complex. However, no previous studies have explored the genomic commonalities and differences among these fungi. In this study, a previously completed genetic linkage map for an interspecific cross between *Fusarium temperatum* and *F. circinatum*, together with genomic sequence data, was utilized to consider the level of synteny between the three *Fusarium* genomes. Regions that are homologous amongst the *Fusarium* genomes examined were identified using *in silico* and pyrosequenced amplified fragment length polymorphism (AFLP) fragment analyses. Homology was determined using BLAST analysis of the sequences, with 777 homologous regions aligned to *F. fujikuroi* and *F. verticillioides*. This also made it possible to assign the linkage groups from the interspecific cross to their corresponding chromosomes in *F. verticillioides* and *F. fujikuroi*, as well as to assign two previously unmapped supercontigs of *F. verticillioides* to probable chromosomal locations. We further found evidence of a reciprocal translocation between the distal ends of chromosome 8 and 11, which apparently originated before the divergence of *F. circinatum* and *F. temperatum*. Overall, a remarkable level of macrosynteny was observed among the three *Fusarium* genomes, when comparing AFLP fragments. This study not only demonstrates how *in silico* AFLPs can aid in the integration of a genetic linkage map to the physical genome, but it also highlights the benefits of using this tool to study genomic synteny and architecture.

## Introduction

Next generation sequencing has resulted in a rapidly accumulating body of genomic data and consequently, genome assemblies are emerging for species representing every form of life (see refs [1], [2]). This is also evident for fungi in the genus *Fusarium*, which includes many important pathogens of plants and species of medical and veterinary importance [3]–[9]. The availability of these whole-genome sequences has allowed unparalleled comparisons between different *Fusarium* species [3]–[5], [8], [9] and significant progress has also been made regarding our understanding of their genomic organization. This is especially true for species associated with disease, where lineage-specific chromosomes have been linked to pathogenicity [3]–[5].

The *Gibberella fujikuroi* complex includes more than 50 *Fusarium* species, many of which represent important plant pathogens [10], [11]. Others are important producers of highly toxic secondary compounds that contaminate global food and feed stocks [12], [13]. Phylogenetic data generally supports the separation of this group of fungi into three large clades, which are thought to reflect the phylogeography of the complex [14], [15]. For example, the clade containing the maize ear rot fungus, *Fusarium verticillioides*, is thought to have African origins, while the clades containing *Fusarium fujikuroi* (the causal agent of bakanae disease in rice seedlings) and *Fusarium circinatum* (the pitch canker pathogen) probably have Asian and American origins, respectively.

Due to their economic importance, various ongoing studies on species in the *G. fujikuroi* complex aim to complement classical mycology and genetic studies with next generation sequencing technologies. The ultimate aim will be to unravel the biological characteristics unique to species in this complex. Significant progress has already been made for the *G. fujikuroi* complex with whole-genome sequence information already available for *F. verticillioides, F. fujikuroi* and *F. circinatum* [6], [7], [9], representing the three phylogeographic clades of this complex. The *F. circinatum* genome is partially assembled and consists of 4145 unordered contigs [6], while the respective *F. verticillioides* and *F. fujikuroi* genome sequences have been ordered into eleven [7] and twelve chromosomes [9]. The shared synteny between *F. fujikuroi* and *F. verticillioides* has also been highlighted recently [9].

Apart from whole-genome sequence data, genetic linkage maps are also available for species in the *G. fujikuroi* complex [16]–[18]. These include genetic linkage maps for *F. verticillioides*, as well as for an interspecific cross between *F. circinatum* and a closely related maize pathogen, *Fusarium temperatum* (S1 Text) [19]–[21]. *Fusarium temperatum* is a member of the American clade and was previously described as a cryptic species of *Fusarium subglutinans* [22]. To fully exploit the availability of these resources it is necessary to integrate information from genetic linkage maps with the physical whole-genome sequence data obtained for the respective species as have been done for other fungi [23], [24]. This would in turn facilitate and advance the use of qualitative trait locus (QTL) analysis for studying the molecular basis of important biological properties.

The primary objective of this study was therefore to integrate the genetic linkage map generated for the *F. circinatum* x *F. temperatum* cross with the available genome sequence data for *F. verticillioides*, *F. fujikuroi* and *F. circinatum* [6], [7], [9]. We also aimed to study the synteny among these genomes and to compare and contrast the genomic architectures of the three species. To achieve these aims, we utilized the available genome sequence information together with analyses of the sequences and sizes of Amplified Fragment Length Polymorphism (AFLP) fragments [25] from *F. circinatum*. The latter involved pyrosequenced AFLP fragments in conjunction with data generated using *in silico* AFLP analyses [26]. Direct genome comparisons using these AFLP fragment sequences allowed for their placement on the three *Fusarium* genomes. This revealed substantial levels of synteny, which in turn allowed for an investigation of the architectural commonalities and specific attributes of the three species.

## Methods

### Analysis of pyrosequenced AFLP fragments

DNA was extracted from the two parents of the interspecific cross [18] between *F. circinatum* (isolate MRC 7870) and *F. temperatum* (isolate MRC 7828) (S1 Text). AFLP fingerprints [25] were generated for the two parents using 3 selective-amplification primer combinations (of the 13 used to generate the genetic linkage map [18]) (Table 1). These three primer combinations were chosen because they provided the best coverage of the twelve linkage groups.

**Table 1:** Summary of pyrosequenced AFLP fragments.

AFLP primer combination^1^	Number of linkage groups covered^2^	AFLP sequences^3^		Unique AFLP sequences^4^		Total number of sequences^5^
		*F. circinatum*	*F. temperatum*	*F. circinatum*	*F. temperatum*	
AA/AA	10	330	300	59	53	84 (98^6^)
CA/TC	12	251	238	77	63	108 (124^6^)
AG/AC	11	254^***^	183^***^	64^***^	21^***^	71 (78^6^)
Subtotal		835*	721*	200^***^	137^***^	
Total		1556		337		263 (300^6^)

1 AFLP primer sequences include two selective nucleotides and are indicated for the respective *Mse*I and *Eco*RI primers.

2 Based on the results of the study conducted by De Vos *et al.* [18].

3 Sequences that carry AFLP adaptors at both ends and that correspond to either *F. circinatum* or *F. temperatum*.

4 AFLP sequences originating from *F. circinatum* and *F. temperatum* after removal of duplicate sequences.

5 After filtering out the homologous sequences (*i.e.* monomorphic bands) present in both *F. circinatum* and *F. temperatum*.

6 Indicated are the total number of pyrosequenced AFLP fragments which includes the 37 monomorphic bands.

^*^Significant deviation between markers originating from *F. circinatum* and *F. temperatum*. Significant deviation is noted as follows: ^*^ 5%, ^**^1% and ^***^0.1%.

Libraries for pyrosequencing on the GS FLX (Roche Diagnostics, Basel, Switzerland) were prepared from the AFLP fragments mixture. However, the usual steps involving DNA fragmenting and “polishing” for subsequent ligation of the ‘A’ and ‘B’ pyrosequencing adaptors were omitted. Instead, PCR was used to add the ‘A’ and ‘B’ pyrosequencing adaptors to the ends of the AFLP fragments, which already carried the same sequences at their 5′ and 3′ ends corresponding to the adaptors and pre-selective PCR primers used for generating the AFLP fragments. Therefore, primers complementary to these ends, and carrying the 19 base pair (bp) pyrosequencing adapter sequences on the 5′ end were synthesized (*Eco*RI + A adaptor: GCCTCCCTCGCGCCATCAGGACTGCGTACCGAATTC and *Mse*I + B adaptor: TTACTCAGGACTCATCCTGGATGAGTCCTGAGTAATTAA). For the PCR, we utilized PyroStart *Taq* polymerase (Fermentas Life Sciences, Ontario, Canada) according to the supplier's protocol as it is suitable when using large primers with very high annealing temperatures. This enzyme is able to extend at a range of temperatures, allowing annealing and extension to take place simultaneously. The two libraries were pyrosequenced using the GS FLX system (Inqaba Biotec, South Africa), although at the time, the length of the fragments that could be completely pyrosequenced ranged from 250–500 bp in length.

Sequences were analyzed and edited using the Vector *NTI ADVANCE* 9.0 software (Invitrogen Life Technologies, Midrand, South Africa). The relative locations of these pyrosequenced fragments within the *F. circinatum* [6], *F. verticillioides* [7] and *F. fujikuroi* [9] genomes were then determined. For this purpose, the sequences were BLAST searched against the genome sequences for these fungi using the ‘Local BLAST’ function of CLC Genomics Workbench (v6.0.1, CLC bio, Denmark) with a significance threshold cutoff E-value of ≤1×10^−10^. The E-value is dependent on the length of the sequence under interrogation, with almost identical short alignments having significant E-values. Therefore, any AFLP fragment that gave ambiguous results was discarded from further analyses. However, sequences displaying the necessary similarity to *F. circinatum*, but not to *F. verticillioides* and *F. fujikuroi*, were subjected to a second round of BLAST. Here, the short length of the query sequence was most often the cause for not finding positive hits in the other two genomes. To circumvent this problem, the length of the pyrosequenced *F. circinatum* fragments were extended using the genome sequence data for this fungus (*e.g.,* a 100 bp fragment that showed similarity to *F. circinatum* but not to the other genomes was increased in length to 300 bp, by including 100 bp sequence data from each of the regions found flanking it in the *F. circinatum* genome). If, however, the longer fragment still did not match any of the sequences in *F. verticillioides* and *F. fujikuroi*, the sequence of the *F. circinatum* fragment was extended a second and final time (the above 300 bp fragment was increased in length to 500 bp by including 100 bp sequence data from each of the flanking regions found in the *F. circinatum* genome) and used in BLAST searches. After, this, if no significant hit was found in BLAST searches to *F. verticillioides* and *F. fujikuroi*, the pyrosequenced AFLP fragment was recorded as showing only a hit to *F. circinatum.*

### *In silico* AFLP fragment analysis and placement on the *F. circinatum* linkage map

*In silico* AFLP fragments were generated for *F. circinatum* by making use of the available genome sequence (this has been deposited at DDBJ/EMBL/GenBank under the accession AYJV00000000, version AYJV01000000) and the program AFLPinSilico (v2, [26]). This was performed for the same 13 primer combinations used to generate the genetic linkage maps for the cross between *F. circinatum* and *F. temperatum* [18]. In this way it was possible to include the sequences for larger AFLP fragments in our subsequent analyses, as well as the sequences for AFLP products associated with the additional 10 primer combinations used for constructing the linkage map. BLAST searches were then performed with these AFLP sequences against the other two *Fusarium* genomes (as described above) to determine their position and presence in these other genomes. The two gene-based markers included on the original genetic linkage map (the mating type idiomorph and histone (H3) gene), as well as three others (translation elongation factor 1-α, β-tubulin and calmodulin, S2 Text) were also linked to the *Fusarium* genomes.

An attempt was made to associate the 148 mapped AFLP markers with known size to their *in silico* counterparts, which in turn would allow assignment of these map markers to actual genomic positions and sequences. As detailed previously, the size of AFLP fragments on the LI-COR gel were estimated using the Saga^MX^ AFLP Analysis Software package [18]. The AFLP adaptors were included in this size estimation, which added an additional 24 bp to the size of the AFLP fragment. Thus, the ‘true’ size of the AFLP fragments on the genetic linkage map, was the size (as indicated) minus 24 bp [18]. When assigning the AFLP map markers to their *in silico* counterparts, possible mistakes made during the initial *in silico* scoring of the gels were also considered [18], by allowing <5 nt variation in the length of the bands scored. This proved appropriate on subsequent comparisons between mapped fragment sizes and *in silico* fragment sizes.

### Genome comparisons

Comparison of the order of genetically mapped AFLP markers of *F. circinatum*, with the locations of homologous sequences from the genomes of *F. verticillioides* and *F. fujikuroi*, was visualized using the program Genome Synteny Viewer (GSV, [27]). Because only a draft *F. circinatum* assembly is available [6], complete genome-wide comparison of this *Fusarium* species to the genome sequences of the two other *Fusarium* species was not possible. However, with the aid of the *in silico* generated AFLP fragments that we associated with their mapped counterparts, it was possible to link the *F. circinatum* map markers of the genetic linkage map [18], to the genomes of *F. verticillioides* and *F. fujikuroi*.

Collinearity between the physical location of markers and their genetic map location was also used as an indication of the level of certainty in placement of markers on the genome sequence of *F. verticillioides* and *F. fujikuroi*. If there was uncertainty over the putative genomic location of an AFLP map marker, a linked marker was used to clarify any ambiguity [28], where that assumption was that two linked map markers would be placed close to each other on the physical genomic sequence. If uncertainty still prevailed, the marker was regarded as not having homology to the specific *Fusarium* species and was not included in further analyses.

### AFLP fragment distribution

We examined the distribution of AFLP fragments on the 11 chromosomes of *F. verticillioides* and *F. fujikuroi*, to determine the coverage of these chromosomes with AFLPs using the 13 primer combinations used. Investigations into all possible AFLP fragments was performed with the program AFLPinSilico (v2, [26]), using no selective nucleotides, to select for the maximum possible number of AFLP fragments per chromosome. This was done to compare the maximum theoretical number of AFLP fragments to the number of AFLP fragments obtained using only 13 AFLP primer combinations.

To further complement synteny analysis between the *F. circinatum* contigs containing pyrosequenced and/or *in silico* generated AFLP fragments, and the genomes of *F. verticillioides* and *F. fujikuoroi*, sequence identity was investigated using the program Geneious v7.0.4 (Biomatters, available from http://www.geneious.com/). The total length of the identical regions were determined and expressed as a percentage of the total length of the *F. circinatum* contig under examination.

## Results

### Analysis of pyrosequenced AFLP fragments

In order to connect the existing genetic linkage map available for the cross between *F. circinatum* and *F. temperatum* [18] to the genome sequence for *F. circinatum* and the two other *Fusarium* species, it was necessary to obtain sequence information for a subset of the markers used to generate the map. Thus, Roche's GS FLX system was used to pyrosequence the AFLP fragments produced by employing three primer combinations that were specifically chosen to allow maximal coverage of all linkage groups (Table 1). Two sets of AFLP fragments were sequenced: one from each of the parental isolates used to construct the genetic linkage map [18]. No genomic sequence data is available for *F. temperatum* at present, so pyrosequencing of AFLP fragments from this parental isolate provided sequence data to use in comparisons to the other *Fusarium* genomes. A total of 6104 sequence reads were obtained with similar numbers of reads from *F. circinatum* (2992) and *F. temperatum* (3112) (Table 1; *t*-test, *P* = 0.12). Removal of sequences not carrying AFLP adaptors at both ends and duplicate sequences yielded 337 AFLP sequences with a bias towards the *F. circinatum* parent (Table 1; *t*-test, *P* = 0.00060). This was despite the fact that there were statistically similar numbers of bands (*t*-test, *P* = 0.58, results not shown) for the two parents on the LI-COR gels. This inconsistency was most likely due to a partial failure of the AFLP procedure for the primer AG/AC combination in *F. temperatum* (Table 1, *t*-test, *P*<0.001 for primer combination AG/AC). The average GC content of the sequences (excluding the added AFLP adapters) was 43.31%. Thirty-seven monomorphic AFLP fragments were shared between *F. circinatum* and *F. temperatum* (Table 1) yielding 263 pyrosequenced AFLP fragments that were unique to either *F. circinatum* or *F. temperatum* out of a total of 300 different pyrosequenced AFLP fragments.

BLAST searches of the 300 pyrosequenced AFLP fragments to the genomes of *F. circinatum*, *F. verticillioides* and *F. fujikuroi* (Table 2) showed that approximately 63% of the fragments shared significant similarity (E≤1×10^−10^), with specific regions in all three *Fusarium* genomes. Forty-nine of the fragments (16.33%) showed no similarity with the sequences in any of the three genomes and are probably unique to *F. temperatum* for which a genome sequence is not yet available. Fifteen percent were unique to *F. circinatum* and homologs of these fragments were not found in either *F. verticillioides* or *F. fujikuroi*. Furthermore, there were 18 fragments (6%) not shared between all three genomes, but only by two of the species, *i.e.*, 12 fragments were similar in sequence in both *F. circinatum* and *F. verticillioides*, and 6 fragments were similar in sequence in only *F. circinatum* and *F. fujikuroi*.

**Table 2:** Pyrosequenced and *in silico* AFLPs showing homology to *F. circinatum*, *F. verticillioides* and *F. fujikuroi*.

AFLP fragments	Total number of sequences	No homology	Homology to all three species	Homology to FC^1^	Homology to FC^1^ and FV^2^	Homology to FC^1^ and FF^3^
AA/AA^4^	98^5^	7	73	11	4	3
CA/TC^4^	124^5^	30	68	20	5	1
AG/AC^4^	78^5^	12	47	14	3	2
Total	300^5^	49	188	45	12	6
*In silico* AFLPs	928	N/A	777	59	43	49

1 Fusarium circinatum (FC).

2 Fusarium verticillioides (FV).

3 Fusarium fujikuroi (FF).

4 Pyrosequenced AFLP fragments.

5 From Table 1.

### *In silico* AFLP fragment analysis and placement on the *F. circinatum* linkage map

A second approach to connect the existing genetic linkage map for the cross between *F. circinatum* and *F. temperatum* [18] to the genome sequences of the three *Fusarium* species [6], [7], [9], was to employ *in silico* generated AFLP fragments [29]. This strategy made it possible to bypass the major limitations associated with analyses of the pyrosequenced AFLP fragments. Firstly, it was possible to examine significantly longer fragments without being restricted to only those in the 250–500 bp range generated by the GS FLX system. Secondly, it was possible to include the fragments associated with additional primer combinations. For the 13 primer combinations used [18] from the *F. circinatum* genome, a total of 928 *in silico* generated AFLP fragments were found that ranged in size from 15 to 3970 bp. BLAST analyses revealed that 777 (83.73%) fragments shared significant similarity with specific regions in the genomes of *F. circinatum, F. verticillioides* and *F. fujikuroi* (Table 2). Six percent were unique to *F. circinatum,* and 92 (9.91%) were shared by only *F. circinatum* and *F. verticillioides* or *F. circinatum* and *F. fujikuroi*.

Using fragment size analyses it was possible to also associate *F. circinatum* AFLP map markers [18] to their corresponding *in silico* counterparts. Of the original 148 map markers [18], 126 (85.14%) of the *F. circinatum* AFLP fragments were assigned to the genome of *F. circinatum*, and the corresponding homologous regions on the genomes of *F. verticillioides* and/or *F. fujikuroi* were found (Table 3). The remaining 22 map markers that were not assigned to the genome may reflect their erroneous inclusion [23] or sequencing errors in the *F. circinatum* genome [30], which prevented their detection using the *in silico* approach. Therefore, sequence-based characterization of these AFLP markers will be dependent on conventional strategies that include excision and direct sequencing of the fragment to determine a location on the genomic sequence.

**Table 3:** AFLPs that could be aligned to the genomic sequences of *F. verticillioides* and *F. fujikuroi*.

Chromosome^1^	Pyrosequenced AFLP fragments	*In silico* generated AFLP fragments	AFLP map markers^2^
1	39	121	12
2	25	86	12^a^
3	24	103	12
4	14	44	8
5	13	96	22
6	14	86	12^b^
7	7	69	14
8	17	41	5^c^+6^d^
9	9	48	10
10	3	33	7
11	11	37	4+5^d^
Total	176^3^	764	129
(12	N/A	8	N/A)

1 Based on the assignments of the *F. verticillioides* genome [7].

2 Map markers that were included in addition to the original data generated by De Vos *et al*. [18] are indicated by a-d in parentheses. Here a, b and c respectively indicate markers for the β-tubulin, translation elongation factor 1-alpha and calmodulin genes (S2 Text), while d indicates AFLP markers for the translocation between chromosome 8 and 11.

3 Twelve pyrosequenced AFLP fragments (of the total of 188) gave ambiguous results when aligned to the genomic sequences of *F. verticillioides* and *F. fujikuroi* and were thus excluded from the dataset. None of these corresponded to AFLP map markers [18].

Due to the fact that *F. verticillioides* was used as the reference genome, only the eleven chromosomes in this particular strain were used, even though members of the *G. fujikuroi* species complex are believed to have twelve chromosomes [17], [31]. For this reason, the designations of the chromosomes of *F. verticillioides* [7] was used to assign contigs of *F. circinatum*, as well as linkage groups from the interspecific cross of *F. circinatum* and *F. temperatum*, to specific chromosomes.

### Synteny

**Figure 1:**
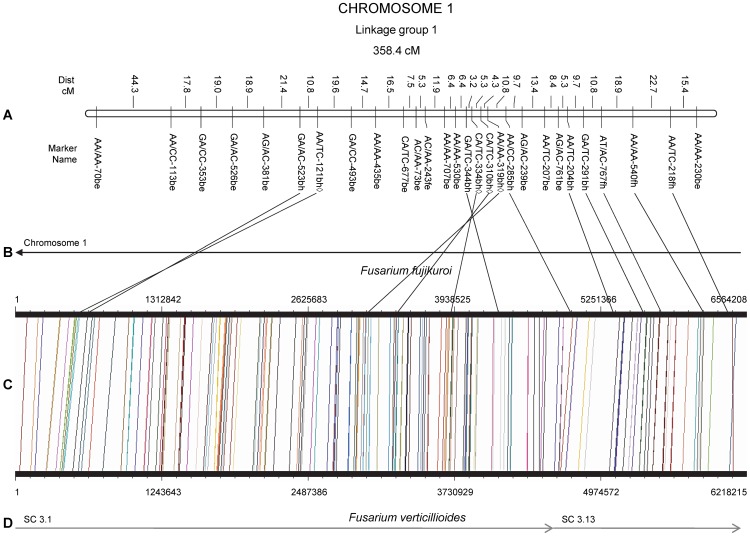
Integration of the genetic linkage map with chromosome 1 of *F. verticillioides* and *F. fujikuroi*. Indicated in (A) is the genetic linkage map between *F. circinatum* and *F. temperatum* [2]. (B) denotes supercontig(s) (sc) of *F. verticillioides* and (D) denotes *F. fujikuroi* chromosomes. Grey supercontigs/chromosomes indicate a forward orientation to what is available, whilst black indicates reverse orientation [3], [4]. (C) designates the syntenous AFLP regions between *F. fujikuroi* and *F. verticillioides*, as indicated by vertical lines. Here, the size (in bp) of the respective chromosomes, are given. Solid lines joining A to C indicate AFLP homologous sequences between the genetic linkage map and *F. fujikuroi* and *F. verticillioides*. Dashed lines indicate synteny between *F. circinatum* and *F. verticillioides* or *F. fujikuroi* (as revealed by comparison of the positions of homologous AFLP fragments). The symbol ◊ after the marker names of the genetic linkage map indicates markers not displaying collinearity.

Comparison of the order of genetically mapped AFLP markers of *F. circinatum* with the locations of homologous sequences from the genomes of *F. verticillioides* and *F. fujikuroi* was visualized using the Genome Synteny Viewer (GSV [27]) (Fig. 1 provides a representative chromosome (1) and S3 Text). However, these comparisons included only those sequences for which homologous regions had been detected in the genomes of *F. verticillioides* and *F. fujikuroi*. Also, three additional gene sequences, translation elongation factor 1-α, β-tubulin and calmodulin (S2 Text), as well as the histone *H3* gene and the mating type (*MAT*) locus already placed on the genetic linkage map [18], were localized to homologous regions in the other *Fusarium* genomes. These analyses revealed that the *F. verticillioides* chromosomes generally aligned with single genetic linkage groups identified previously for the interspecific cross between *F. circinatum* and *F. temperatum* [18].

We also visualized the 777 *in silico* generated AFLP fragments for *F. circinatum* that showed homology to *F. verticillioides* and *F. fujikuroi* with GSV [27] (Fig. 1 and S3 Text). From these visualizations (where homologous AFLP fragment positions were indicated by vertical lines between the *F. verticillioides* supercontigs and *F. fujikuroi* chromosomes) it was clear that there was a great deal of synteny between *F. verticillioides* and *F. fujikuroi*.

These comparisons also allowed the probable assignment of two additional unmapped supercontigs from *F. verticillioides* [7] to chromosomes. These are supercontig (sc) 3.30 that was added to chromosome 6 and sc 3.33 that was added to chromosome 7 (S3 Text). Here, we assumed that the chromosomal organization between *F. verticillioides* and *F. fujikuroi* is highly similar, as described previously [9]. However, additional work is required to confirm our assignment of these unmapped supercontigs of *F. verticillioides* to chromosomes.

Furthermore, eight *F. circinatum in silico* generated AFLP fragments showed homology to the twelfth chromosome of *F. fujikuroi* [9], suggesting that either *F. circinatum* has a twelfth chromosome or that there are sequences homologous to this chromosome. The latter markers showed no homology to *F. verticillioides*, which is in agreement with the fact that the strain of *F. verticillioides* sequenced, lacked the twelfth chromosome and contained no genetic markers in the sequence data for this chromosome [4].

While there was overall synteny between *F. verticillioides* and *F. fujikuroi*, there was a size discrepancy between chromosome 4 in *F. fujikuroi* (3301440 bp) and *F. verticillioides* (4234339 bp) (S3 Text). This finding that the *F. fujikuroi* chromosome is *ca*. 0.9 Mbp smaller than the corresponding *F. verticillioides* chromosome, has been reported previously [9] and our data support the finding that this size difference is due to two large regions that were lost from the *F. fujikuroi* chromosome relative to the *F. verticillioides* chromosome. Furthermore, chromosome 4 in *F. verticillioides* and *F. fujikuroi* corresponds to two linkage groups (LG 12 and 10) from the genetic linkage map generated for the interspecific cross between *F. circinatum* and *F. temperatum* [18]. Additionally, we detected genomic rearrangements between *F. verticillioides* and *F. fujikuroi.* One of these, presented as a large inversion on chromosome 11 (S3 Text), had been reported previously [9]. In addition, we also detected several smaller insertions/deletions (Fig. 1 and S3 Text).

**Figure 2:**
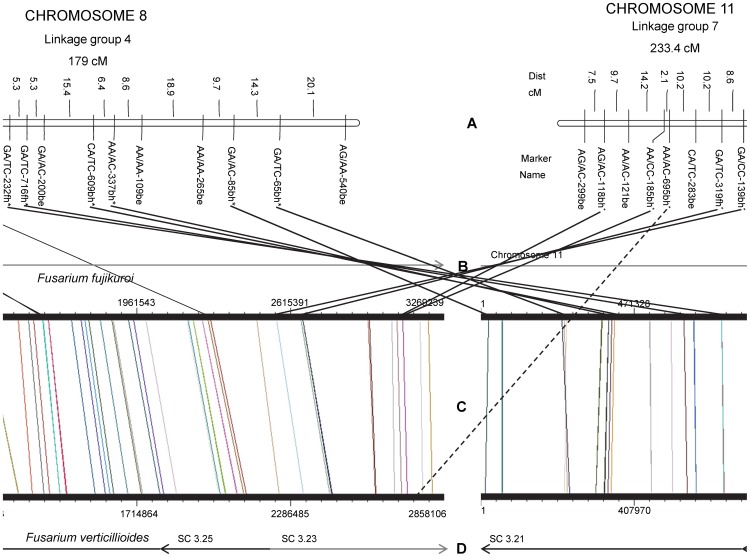
Reciprocal translocation between chromosomes 8 and 11. Indicated in (A) is the genetic linkage map between *F. circinatum* and *F. temperatum* [2]. (B) denotes supercontig(s) (sc) of *F. verticillioides* and (D) denotes *F. fujikuroi* chromosomes. Grey supercontigs/chromosomes indicate a forward orientation to what is available, whilst black indicates reverse orientation [3], [4]. (C) designates the synteny between *F. fujikuroi* and *F. verticillioides*, as indicated by vertical lines. Here, the size (in bp) of the respective chromosomes, are given. Solid lines joining A to C indicate AFLP homologous sequences between the genetic linkage map and *F. fujikuroi* and *F. verticillioides*. Dotted lines are indicative of synteny between the genetic linkage map and either *F. fujikuroi* or *F. verticillioides*. In chromosome 8 and 11, asterisks indicate those *F. circinatum* markers involved in the reciprocal translocation.

Comparison of the *F. circinatum* genetic linkage groups to the chromosomes of *F. verticillioides* and *F. fujikuroi* revealed a potential reciprocal translocation between the distal ends of chromosome 8 and 11 (Fig. 2). This was evident from six *F. circinatum* markers in LG 4 and five in LG 7 (S3 Text), which aligned respectively to chromosomes 8 and 11, of *F. verticillioides* and *F. fujikuroi*. These markers were inverted relative to the genomes of both *F. verticillioides* and *F. fujikuroi* (Fig. 2). To test whether a miscalculation had occurred in the original construction of LG 4 and LG 7, the original data were reanalyzed in MapMaker, which confirmed that these two linkage groups are independent, with no markers showing linkage between them.

To determine whether the translocation was present in the parents, we investigated the genetics of the *F. circinatum* x *F. temperatum* cross (S4 Text). With the progeny of the *F. circinatum* and *F. temperatum* cross, we found that in 82.98% of the progeny the translocation markers were recombinant for the *F. circinatum* and *F. temperatum* alleles [18]. This strongly suggests that it was present in both the *F. circinatum* and *F. temperatum* parents of the interspecific cross used to generate the genetic linkage map (S4 Text).

### AFLP fragment distribution

The distribution of AFLPs on chromosomes was found to be 25.43±5.00 AFLP homologous markers per 1Mbp of sequence for *F. verticillioides*, which was not statistically different (*t*-test, *P* = 0.40) to that observed for *F. fujikuroi* (23.72±4.37 per 1 Mbp). However, chromosome 4 had less homologous markers per Mb than expected in both *F. verticillioides* (Poisson distribution, *P* = 0.0027) and *F. fujikuroi* (Poisson distribution, *P* = 0.016), due to the missing 0.9 Mbp of sequence in the *F. fujikuroi* assembly. In addition, fewer markers were mapped to chromosome 10 in *F. fujikuroi* (Poisson distribution, *P* = 0.023).

Examination of all possible AFLP fragments (excluding the selective nucleotides) on the genomic sequence data revealed that by using 13 primer combinations, there are 7.98±3.36 (per 1 Mbp of sequence) times fewer AFLP markers in *F. verticillioides*, and 17.17±3.39 (per 1 Mbp of sequence) times fewer AFLP markers in *F. fujikuroi*, when compared to results using all possible AFLP markers. Therefore, the distribution of markers over the chromosomes (from this study) was not statistically different between *F. verticillioides* and *F. fujikuroi*, despite the fact that there was a bias towards *F. verticillioides* having less AFLP markers (with no selective nucleotides) than *F. fujikuroi*. This large difference in possible AFLP fragments in *F. fujikuroi* and *F. verticillioides*, was unexpected and unlikely to be associated with restriction enzymes employed for generating the fragments. Both *Eco*RI and *Mse*I are AT-rich cutters, but the %AT is similar in both *F. fujikuroi* (52.5%) and *F. verticillioides* (51.3%). Nevertheless, the chromosomal distribution of the AFLP markers associated with these 13 primer combinations was not statistically different between *F. verticillioides* and *F. fujikuroi*.

The pyrosequenced and *in silico* generated AFLP fragments were distributed over 718 contigs of *F. circinatum* (S5 Text). This represents 17.32% of the total of 4145 contigs [6]. In some cases, more than one marker sequence was found on a contig. These contigs equate to 19 657 701 bp, or 44.71% of the estimated genome size of *F. circinatum* [6], which reflects the fact that only 13 primer combinations were used for the *in silico* AFLP fragment analysis.

Examination of the synteny of the 718 contigs (which includes 45% of the total genome) of *F. circinatum* to the genomes of *F. verticillioides* and *F. fujikuroi*, was done by looking at the sequence identity between them (S5 Text). This revealed an average sequence identity of 86.80% for *F. circinatum* and *F. verticillioides* and 85.43% sequence identity for *F. circinatum* and *F. fujikuroi*. Additionally, there was a significant difference between the sequence identity for chromosome 4 of *F. circinatum* when compared to *F. verticillioides* and *F. fujikuroi*. This is due to the size difference of this chromosome in *F. fujikuroi*, with *ca.* 0.9 Mbp missing.

## Discussion

In this study, sequence-based characterized AFLP fragments were used to directly compare the genomes of three *Fusarium* species [6], [7], [9] each of which represent one of the phylogeographic clades [14], [15] in the *G. fujikuroi* complex. The emerging data confirmed the remarkable levels of synteny previously found between *F. verticillioides* and *F. fujikuroi* [9], and that this pattern of conservation would most likely be extended throughout the *G. fujikuroi* complex. Integration of genetic linkage data [18] with the genomic assemblies using AFLPs enabled exploration of the chromosomal arrangements of these *Fusarium* species. Using this, we found evidence for a reciprocal chromosomal translocation in *F. circinatum* and *F. temperatum*, two closely related species that are able to hybridize [19].

Anchoring of a genetic linkage map to genomic sequence data provides a powerful tool, particularly to localize important genetic markers to physical genome sequences [29]. The genetic linkage map used in this study was constructed primarily with anonymous AFLP markers [18]. In contrast to the more conventional approach used to assign sequence data to such markers, which usually involves excision of AFLP bands from gels and subsequent cloning and sequencing, the *in silico* approach to convert anonymous AFLP markers into sequence-based characterized markers [28], [29] is much less labour intensive and time consuming. Following this computer-based procedure, 85% of the *F. circinatum* AFLP markers were placed on the genomic sequences of *F. circinatum, F. verticillioides* and/or *F. fujikuroi*.

The reason for considering pyrosequenced AFLP fragments in conjunction with *in silico* generated AFLP fragments was two-fold. This approach allowed us, firstly, to assign AFLP map markers/linkage groups to genomic sequence data, and secondly, to determine the extent of the synteny amongst the three *Fusarium* genomes. Apart from aiding in directly linking the *F. circinatum* genome sequence to the genetic linkage map for this fungus, analyses of the pyrosequenced AFLP fragments also provided information on *F. temperatum*. Therefore, despite the lack of genome sequence information, data generated by pyrosequencing of the AFLP fragments allowed genome comparisons of *F*. *temperatum* to the other *Fusarium* genomes. However, the use of *in silico* generated AFLP fragments allowed us to expand our analyses to AFLP fragments of any length and not only those in the size range 40–1000 bp that are typically evaluated when using gel electrophoresis. The use of *in silico* AFLPs (when having a genome sequence available) is, therefore, an extremely cost-effective alternative to sequencing individual AFLP bands, in linking the sequence and placement of AFLP markers to genomic sequence.

Collinearity between the physical location of markers (to the genomes of *F. verticillioides* and *F. fujikuroi*) and their location on the *F. circinatum* x *F. temperatum* genetic linkage map generally corresponded well. The most apparent exception to collinearity was the reciprocal translocation observed on chromosome 8 and 11 of the linkage map, which was inverted relative to *F. verticillioides* and *F. fujikuroi*. Of the 129 markers examined, only 18 (13.18%) appeared to be located at unexpected positions or orientations in one or more of the genomes and were represented by inversions between two or three adjacent AFLP map markers on chromosomes 1, 5, 6, 7 and 9. In comparison, Gale et al. (2005) showed that only 1.28% of all map markers on the *Fusarium graminearum* genetic linkage map did not correspond to their genomic locations [23]. The difference between the two studies is probably the result of the interspecific nature of the mapping population considered in the current study. It has previously been shown that the F_1_ progeny of the *F. circinatum* x *F. temperatum* cross displayed a preferential inheritance of alleles as well as intact chromosomes from *F. temperatum* [18]. Also, 55% of the mapped markers included in this study displayed segregation distortion (*P*<0.05) [18]. Such high levels of segregation distortion of alleles or map markers are known to significantly complicate the inference of marker/allele order when compiling genetic linkage maps [32], [33].

A significant proportion of the sequence-characterized AFLP fragments appeared to be unique to a specific species. For the pyrosequenced AFLP fragments from *F. circinatum* and *F. temperatum*, the results showed that 16.33% of the fragments had no similarity to any of the genomic sequences in the three sequenced *Fusarium* species. Although the generally short length of these fragments might have influenced their BLAST-based identification, a large proportion of these sequences were probably unique to *F. temperatum*, for which genome sequences are not available. This is consistent with the fact that 15% of the pyrosequenced AFLP fragments were present only in *F. circinatum*. This overall trend was also observed with the *in silico* sequence-characterized AFLP fragments, where 6.35% appeared to be unique to *F. circinatum*. This lower value is the result of additional rounds of BLAST whereby the size of the fragment was increased substantially up- and downstream to resolve ambiguous results. Additionally, in our analysis of a subset of the total *F. circinatum* contigs harbouring AFLP fragments, approximately 13–15% of the *F. circinatum* genome shares no sequence identity to either *F. verticillioides* or *F. fujikuroi*. Previous studies have shown that *Fusarium* species have a core genome that appears to be shared among species and that a proportion of the genome might be unique to a specific species [4]. For example, synteny studies showed that there is approximately 10% unique genomic sequence between *Fusarium oxysporum* f. sp. *lycopersici* and *F. verticillioides*, and 20% between *F. oxysporum* f. sp. *lycopersici* and *F. graminearum*. Although additional work is required, our data thus suggests that these unique AFLP regions are indeed representative of unique genomic regions of species within the *G. fujikuroi* complex.

Based on the known karyotypes for some species in the *G. fujikuroi* complex, it is thought that species in this group have twelve chromosomes [31]. In *F. verticillioides* and *F. fujikuroi*, the twelfth chromosome was found to be meiotically unstable (the sequenced strain of *F. verticillioides* harboured only eleven chromosomes [7]), duplicated and deleted in other strains, and also to have low sequence homology [17], [31]. In *F. fujikuroi* it has been shown that this dispensable supernumerary chromosome is not required for pathogenicity, because those isolates not containing this chromosome retained their pathogenicity [9]. The twelfth chromosome further appears to be subject to size variation, and its presence in the *G. fujikuori* complex, strain-specific [9], [31]. In this study, we found that the twelve linkage groups for the interspecific cross between *F. circinatum* and *F. temperatum* [18] corresponded only to the eleven chromosomes of the sequenced *F. verticillioides* strain [7], and the first 11 chromosomes of *F. fujikuroi* [9]. However, eight of the *in silico* generated AFLP fragments for *F. circinatum* showed homology to chromosome 12 of *F. fujikuroi*, but not to any of those of *F. verticillioides*. This suggests that *F. circinatum* does have 12 chromosomes or that the *F. circinatum* genome has sequences homologous to the twelfth chromosome in *F. fujikuroi*. It was not possible to confirm the presence of these homologous sequences or chromosome 12 in *F. temperatum* and/or the F_1_ progeny of the interspecific cross, using the available data [18].

A putative reciprocal translocation was observed on the genetic linkage map of the interspecific cross between *F. circinatum* and *F. temperatum*. This region was found to be translocated relative to the genomic sequence of both *F. verticillioides* and *F. fujikuroi*. A similar translocation has previously been reported in *Fusarium oxysporum* f. sp. *lycopersici* relative to *F. verticillioides* [4]. Such chromosomal translocations have also been documented in other fungal species. For example, in *Neurospora crassa*, crosses between normal strains and strains harbouring translocations can yield progeny with duplications that disrupt genes and create novel open reading frames [34]. *Cryptococcus neoformans* var. *grubii* contains a chromosomal translocation, not present in two other varieties of the pathogen, which disrupts two genes that affect numerous virulence factors [35]. Our study represents the first report of a chromosomal translocation within the *G. fujikuroi* complex. The question remains as to whether the detected translocation event represents an ancestral or a recent state. Based on the genetics of *F. circinatum* and *F. temperatum*, the translocation must have been present in both the parental isolates. A translocation that is present in both *F. circinatum* and *F. temperatum*, but not *F. verticillioides* or *F. fujikuroi*, would further imply that the translocation event is ancestral to the divergence of *F. circinatum* and *F. temperatum*. Some authors have noted that fixed translocations between homologous chromosomes represent a barrier to gene flow [36], which suggests that chromosomal translocation could have contributed to speciation in this complex.

Macrosynteny represents the synteny of genes at the chromosomal level, with the backbone of genes on a chromosome being collinear [37]. Synteny was found when comparing *F. oxysporum* f. sp. *lycopersici* with *F. verticillioides*, except for one chromosomal translocation and a few rearrangements [4]. Similar synteny was also found when comparing *F. verticillioides* to *F.fujikuroi* [9]. For the purposes of the current study, we consider macrosynteny as the conservation of genomic sequences (AFLP sequences) and order. Accordingly, we have shown that conservation of AFLP fragments from *F. circinatum* and *F. temperatum* follows a pattern of macrosynteny similar to that observed between *F. verticillioides* and *F. fujikuroi*.

## Conclusions

Homologous AFLP fragments originating from *F. circinatum* were used to show genomic conservation within species in the *G. fujikuroi* complex. Only one reciprocal translocation was found in *F. circinatum* and *F. temperatum*, compared to *F. verticillioides* and *F. fujikuroi*. A few genomic rearrangements between *F. verticillioides* and *F. fujikuroi* were also detected with only one large inversion on chromosome 11. This high level of macrosynteny is thus characteristic of species in the *G. fujikuroi* complex. Knowledge of this macrosynteny will aid future genome assemblies and *in silico* identification of genes of interest in other *Fusarium* species in the complex (*e.g.* [38]). This study has also served to illustrate the usefulness of previously anonymous AFLP fragments from a genetic linkage map, together with a genomic sequence, in synteny analyses between species in the *G. fujikuroi* complex.

## Supporting Information

S1 Text **Confirmation of** ***F. temperatum*** **as a parental isolate for the genetic linkage map generated.**
(DOCX)

S2 Text **PCR-RFLP analysis of three gene regions in** ***F. circinatum*** **and** ***F. temperatum*** **.**
(DOCX)

S3 Text **Integration of the genetic linkage map with the chromosomes of** ***F. verticillioides*** **and** ***F. fujikuroi*** **.**
(DOCX)

S4 Text **The origin of the reciprocal translocation.**
(DOCX)

S5 Text ***F. circinatum*** **sequence identity to** ***F. verticillioides*** **and** ***F. fujikuroi.***
(DOCX)
