# Supplementary material for: Genome-Wide Macrosynteny among Fusarium Species in the Gibberella fujikuroi Complex Revealed by Amplified Fragment Length Polymorphisms
Source: PLoS One. 2014 Dec 8;9(12):e114682. doi: 10.1371/journal.pone.0114682 (PMC4259476; doi:10.1371/journal.pone.0114682)
Supplement: S1 Text — Confirmation of F. temperatum as a parental isolate for the genetic linkage map generated. (DOCX) [file pone.0114682.s001.docx]

**Supporting information file 1**

**Confirmation of *F. temperatum* as a parental isolate for the genetic linkage map generated by De Vos *et al*. [**[**1**](#_ENREF_1)**]**

Previous studies have shown that *F. subglutinans* represents at least two distinct clades or cryptic species [[2-4](#_ENREF_2)]. One of these cryptic species is conspecific with *F. subglutinans* sensu stricto [5], while the other was elevated to species level and formally described as *Fusarium temperatum* [[5](#_ENREF_5)]. However, the 2002 [5] and 2011 [6] studies utilized different sets of isolates. In the 2002 study, the cryptic species corresponding to *F. temperatum* included isolate MRC 7828 (then designated as *F. subglutinans*) that was not included in the 2011 study. This isolate was subsequently used in a hybrid cross with *F. circinatum* to generate a genetic linkage map for these fungi [1]. Here, we wanted to demonstrate that isolate MRC 7828 represents *F. temperatum*, and not *F. subglutinans*, by making use of a phylogenetic approach. For this purpose we used DNA sequences for the genes encoding translation elongation factor 1-α and β-tubulin (BT) [[6](#_ENREF_6), [7](#_ENREF_7)]. Sequence alignments included data for isolate MRC 7828 together with all of the isolates used in the *F. temperatum* description [[5](#_ENREF_5)] and were constructed with MAFFT version 5.85 [[8](#_ENREF_8), [9](#_ENREF_9)]. The best-fit substitution model (TIM2 substitution model with gamma correction [[10](#_ENREF_10)]) was determined with jModeltest [[11](#_ENREF_11)]. Maximum likelihood (ML) phylogenetic analysis was conducted with PhyML version 2.4.3 [[12](#_ENREF_12)] where branch support was estimated using 1000 bootstrap replicates. The resulting phylogenetic tree confirmed that isolate MRC 7828 used as the female parent in the interspecific cross [[13](#_ENREF_13)] indeed represents *F. temperatum* (Supplemental Figure 1).

REFERENCES

1. De Vos L, Myburg AA, Wingfield MJ, Desjardins AE, Gordon TR, (2007) Complete genetic linkage maps from an interspecific cross between *Fusarium circinatum* and *Fusarium subglutinans.* Fungal Genet Biol 44: 701-714.

2. O'Donnell K, Nirenberg HI, Aoki T, Cigelnik E (2000) A multigene phylogeny of the *Gibberella fujikuroi* species complex: Detection of additional phylogenetically distinct species. Mycoscience 41: 61-78.

3. Steenkamp ET, Wingfield BD, Coutinho TA, Wingfield MJ, Marasas WFO (1999) Differentiation of *Fusarium subglutinans* f. sp. *pini* by histone gene sequence data. Appl Environ Microbiol 65: 3401-3406.

4. Steenkamp ET, Coutinho TA, Desjardins AE, Wingfield BD, Marasas WFO, (2001) *Gibberella fujikuroi* mating population E is associated with maize and teosinte. Mol Plant Pathol 2: 215-221.

5. Scauflaire J, Gourgue M, Munuat F: *Fusarium temperatum* sp. nov. from maize, an emergent species closely related to *Fusarium subglutinans.* Mycologia 103: 586-597.

6. O’Donnell K, Kistler HC, Cigelnik E, Ploetz RC (1998) Multiple evolutionary origins of the fungus causing Panama disease of banana: Concordant evidence from the nuclear and mitochondrial gene genealogies. P Natl Acad Sci USA 95: 2044-2049.

7. O’Donnell K, Cigelnik E (1997) Two divergent intragenomic rDNA ITS2 types within a monophyletic lineage of the fungus *Fusarium* are nonorthologous. Mol Phylogenet Evol 7: 103-116.

8. Kazutaka K, Kazuharu M, Kei-ichi K, Takashi M (2002) MAFFT: A novel method for rapid multiple sequence alignment based on fast Fourier transform. Nucl Acids Res 30: 3059-3066.

9. Katoh K, Kuma K-I, Toh H, Miyata T (2005) MAFFT Version 5: Improvement in accuracy of multiple sequence alignment. Nucl Acids Res 33: 511-518.

10. Tavare S (1986) Some probabilistic and statistical problems in the analysis of DNA sequences. Lect Math Life Sci 17: 57-86.

11. Posada D (2008) jModelTest: Phylogenetic model averaging. Mol Biol Evol 25: 1253-1256.

12. Guidon S, Gascuel O (2003) A simple, fast and accurate algorithm to estimate large phylogenies by maximum likelihood. Syst Biol 52: 696-704.

13. Desjardins AE, Plattner RD, Gordon TR (2000) *Gibberella fujikuroi* mating population A and *Fusarium subglutinans* from teosinte species and maize from Mexico and Central America. Mycol Res 104: 865-872.


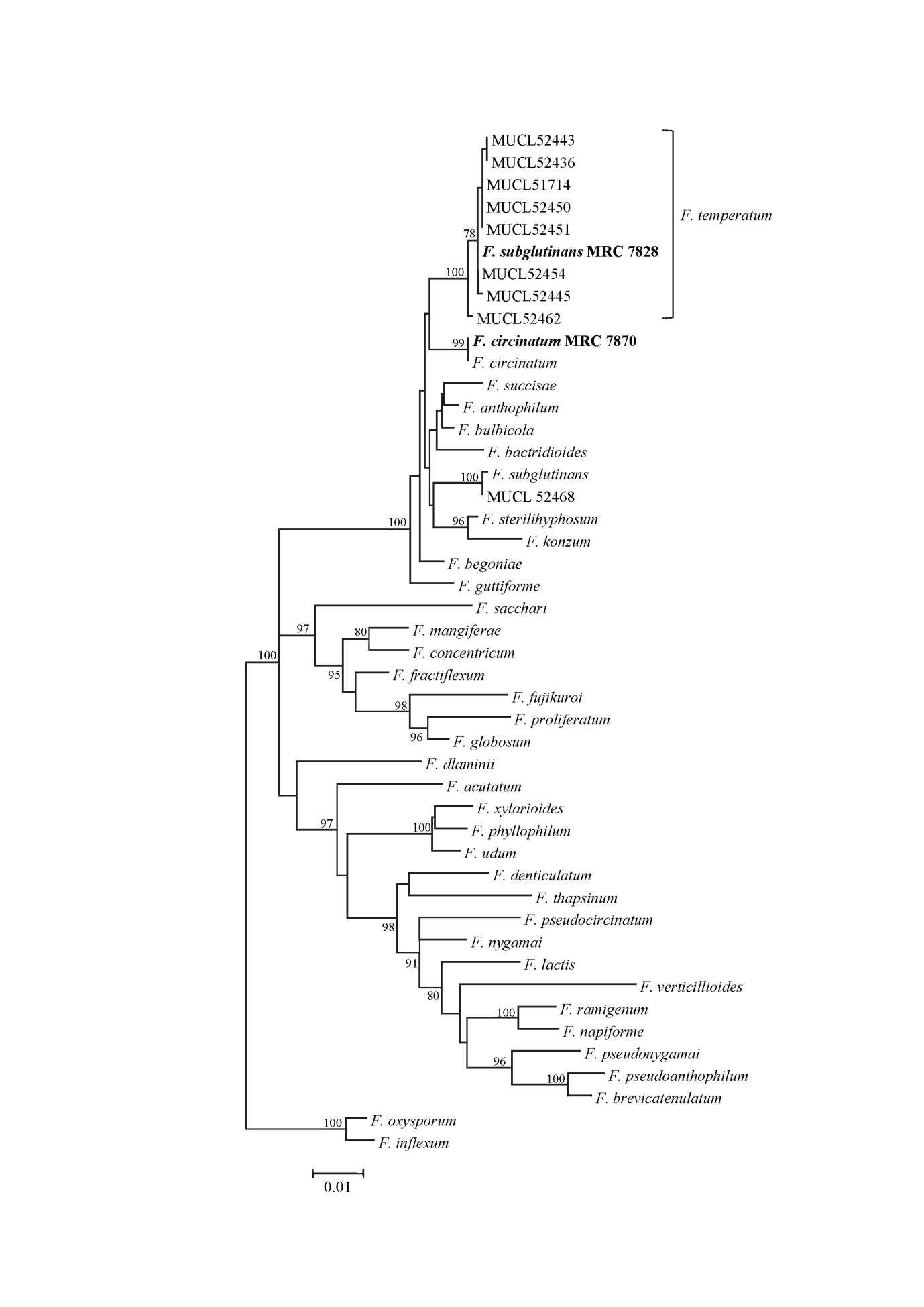


Figure S1. Maximum likelihood tree based on partial translation elongation factor 1-α and the β-tubulin gene sequences. Values at branch nodes are the bootstrapping confidence values with those ≥ 85% shown. Indicated in bold are the *F. circinatum* and *F. temperatum* isolates used to generate the hybrid progeny from which the genetic linkage map was generated by De Vos et al. [1]. For information regarding all other *Fusarium* species and isolates see [5].
