# Supplementary material for: Genome-Wide Macrosynteny among Fusarium Species in the Gibberella fujikuroi Complex Revealed by Amplified Fragment Length Polymorphisms
Source: PLoS One. 2014 Dec 8;9(12):e114682. doi: 10.1371/journal.pone.0114682 (PMC4259476; doi:10.1371/journal.pone.0114682)
Supplement: S2 Text — PCR-RFLP analysis of three gene regions in F. circinatum and F. temperatum . (DOCX) [file pone.0114682.s002.docx]

**Supporting information file 2**

**PCR-RFLP analysis of three gene regions in *F. circinatum* and *F. temperatum*.**

PCR amplification of three gene regions was performed using the DNAs extracted from the two parental isolates. Part of the translation elongation factor 1-α gene was amplified with primers EF-1 and EF-2 [[1](#_ENREF_1)], while the β-tubulin gene region was amplified using primers T1 and T2 [[2](#_ENREF_2)]. Both these gene regions were amplified using previously described conditions [[3](#_ENREF_3)]. A portion of the calmodulin gene was amplified as described before using primers CL1 and CL2A [[4](#_ENREF_4), [5](#_ENREF_5)]. PCR amplicons were purified with ethanol precipitation [[6](#_ENREF_6)] and cloned into the pGEM^®^-T Easy vector (Promega, Madison, WI) according to the manufacturer’s instructions. Sequencing was performed using the T7 and SP6 primers and the ABI PRISM BigDye Terminator v3.0 Cycle Sequencing Kit (Applied Biosystems, Foster City, CA) on a 3730 DNA Analyzer. Sequences were analyzed on BioEdit v7.0.5.3 [[7](#_ENREF_7)] to identify restriction enzymes that will allow differentiation between the two parental alleles for the three genes (Supplemental Table 1). These enzymes were subsequently used in a standard PCR-RFLP procedure to screen the 94 F_1_ progeny to determine the parental origin of the parental alleles. The results were scored as ‘0’ for band absent and ‘1’ for band present.

The PCR-RFLP dataset was imported, together with the original framework linkage marker set [[8](#_ENREF_8)], into MapMaker MACINTOSH v2.0 [[9](#_ENREF_9)]. The 3 gene regions were distributed into linkage groups using an LOD of 9.0, as the dataset separated into 12 linkage groups at this threshold. Using the ‘First Order” command, the most probable placement of each gene was determined. Using MapMaker, EF was placed on linkage group 3, β-tubulin on linkage group 8 and calmodulin on linkage group 4 [[8](#_ENREF_8)].

Placement of the β-tubulin (chromosome 2) and translation elongation factor 1-α (chromosome 6) genes on the linkage map resulted in these markers not displaying collinearity. These were accordingly added using the ‘Group’ command in MAPMAKER, to assign a linkage group to these markers, and ‘First Order’ step to attain a starting order. No further statistical analysis was done and could be a possible reason for the discrepancy of the order of these genes on the linkage maps.

REFERENCES

1. O'Donnell K, Kistler HC, Cigelnik E, Ploetz RC (1998) Multiple evolutionary origins of the fungus causing Panama disease of banana: Concordant evidence from nuclear and mitochondrial gene genealogies. P Natl Acad Sci USA 95: 2044-2049.

2. O'Donnell K, Cigelnik E (1997) Two divergent intragenomic rDNA ITS2 types within a monophyletic lineage of the fungus *Fusarium* are nonorthologous. Mol Phylogenet Evol 7: 103-116.

3. Geiser DM, Ivey MLL, Hakiza G, Juba JH, Miller SA (2005) *Gibberella xylarioides* (anamorph: *Fusarium xylarioides*), a causative agent of coffee wilt disease in Africa, is a previously unrecognized member of the *G. fujikuroi* species complex. Mycologia 97: 191-201.

4. O'Donnell K, Nirenberg HI, Aoki T, Cigelnik E (2000) A multigene phylogeny of the *Gibberella fujikuroi* species complex: Detection of additional phylogenetically distinct species. Mycoscience 41: 61-78.

5. White TJ, Bruns T, Lee S, Taylor J (1990) Amplification and direct sequencing of fungal ribosomal RNA genes for phylogenetics. In PCR protocols: A guide to methods and applications. Edited by Innis MA, Gelfond DH, Sninsky JJ, White TJ. San Diego, California: Academic Press, Inc. p. 315-322.

6. Sambrook J, Fritsch EF, Maniatis T (1989) Molecular cloning: A laboratory manual. New York, USA: Cold Spring Harbor Laboratory Press.

7. Hall TA (1999) BioEdit: a user-friendly biological sequence alignment editor and analysis program for Windows 95/98/NT. Nucl Acid S, 41: 95-98.

8. De Vos L, Myburg AA, Wingfield MJ, Desjardins AE, Gordon TR, et al. (2007) Complete genetic linkage maps from an interspecific cross between *Fusarium circinatum* and *Fusarium subglutinans*. Fungal Genet Biol 44: 701-714.

9. Lander ES, Green P, Abarahamson J, Barlow A, M.J. D, Lincoln SE, et al. (1987) MAPMAKER: An interactive computer package for constructing primary genetic linkage maps of experimental and natural populations. Genomics 1: 174-181.

Supplemental Table 1. PCR-RFLPs of three gene regions in *F. circinatum* and *F. temperatum*.

| Gene | Amplicon size in base pairs | | Restriction enzyme | Restriction fragment size in base pairs | |
| --- | --- | --- | --- | --- | --- |
|  | FC^1^ | FT^2^ |  | FC^1^ | FT^2^ |
| Translation elongation factor 1-α | 706 | 705 | *Dde*I | 416, 182, 55, 53 | 276, 182, 139, 55, 53 |
| β-tubulin | 605 | 604 | *Nla*IV | 350, 147, 84, 24 | 276, 147, 84, 73, 24 |
| Calmodulin | 756 | 754 | *Cfo*I | 308, 282, 164, 2 | 446, 308 |

^1^Size of fragment(s) originating from *F. circinatum* (FC).

^2^Size of fragment(s) originating from *F. temperatum* (FT).
