# Supplementary material for: Genome-Wide Macrosynteny among Fusarium Species in the Gibberella fujikuroi Complex Revealed by Amplified Fragment Length Polymorphisms
Source: PLoS One. 2014 Dec 8;9(12):e114682. doi: 10.1371/journal.pone.0114682 (PMC4259476; doi:10.1371/journal.pone.0114682)
Supplement: S3 Text — Integration of the genetic linkage map with the chromosomes of F. verticillioides and F. fujikuroi . (DOCX) [file pone.0114682.s003.docx]

**Supporting information file 3**

**Integration of the genetic linkage map with the chromosomes of *F. verticillioides* and *F. fujikuroi*.**

Comparison of the order of genetically mapped AFLP markers of *F. circinatum*, with the locations of homologous sequences from the genomes of *F. verticillioides* and *F. fujikuroi*, was visualized using the program Genome Synteny Viewer (GSV [1]) (Supplemental Figure 1-11).

REFERENCES

1. Revanna KV, Chiu C-C, Bierschank E, Dong Q (2011) GSV: A web-based genome synteny viewer for customized data. BMC Bioinformatics 12: 316.

2. De Vos L, Myburg AA, Wingfield MJ, Desjardins AE, Gordon TR, et al. (2007) Complete genetic linkage maps from an interspecific cross between *Fusarium circinatum* and *Fusarium subglutinans*. Fungal Genet Biol 44: 701-714.

3. *Fusarium* Comparative Sequencing Project. Broad Institute of Harvard and MIT. Available: <http://www.broad.mit.edu>. Assessed: June 2013.

4. Wiemann P, Sieber CMK, Von Bargen KW, Studt L, Niehaus E-M, et al. (2013) Unleashing the cryptic genome: Genome-wide analyses of the rice pathogen *Fusarium fujikuroi* reveal complex regulation of secondary metabolism and noval metabolites. PLoS Pathog 9: e1003475.


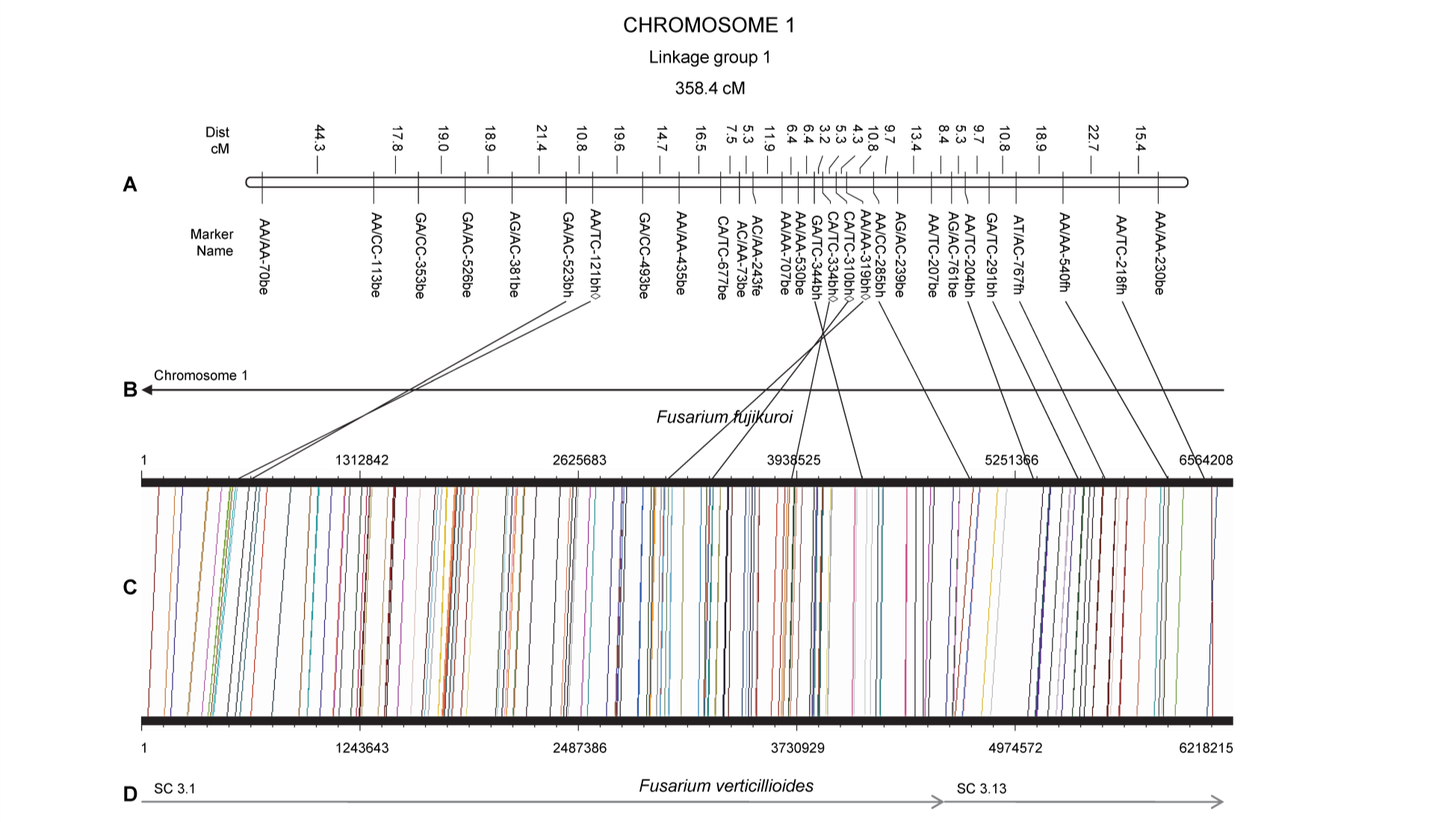


Figure S1.

**Figure S1-S11. Integration of the genetic linkage map with the chromosomes of *F. verticillioides* and *F. fujikuroi*.** Indicated in (A) is the genetic linkage map between *F. circinatum* and *F. temperatum* [2]. (B) denotes supercontig(s) (sc) of *F. verticillioides* and (D) denotes *F. fujikuroi* chromosomes. Grey supercontigs/chromosomes indicate a forward orientation to what is available, whilst black indicates reverse orientation [3, 4]. (C) designates the syntenous AFLP regions between *F. fujikuroi* and *F. verticillioides*, as indicated by vertical lines. Here, the size (in bp) of the respective chromosomes, are given. Solid lines joining A to C indicate AFLP homologous sequences between the genetic linkage map and *F. fujikuroi* and *F. verticillioides*. In Fig S4, red circles indicate the regions present in *F. verticillioides*, but missing in *F. fujikuroi*. In Fig S8 and S11, asterisks indicate those *F. circinatum* markers involved in the reciprocal translocation. Dashed lines indicate synteny between *F. circinatum* and *F. verticillioides* or *F. fujikuroi* (as revealed by comparison of the positions of homologous AFLP fragments). The symbol ◊ after the marker names of the genetic linkage map indicates markers not displaying collinearity.


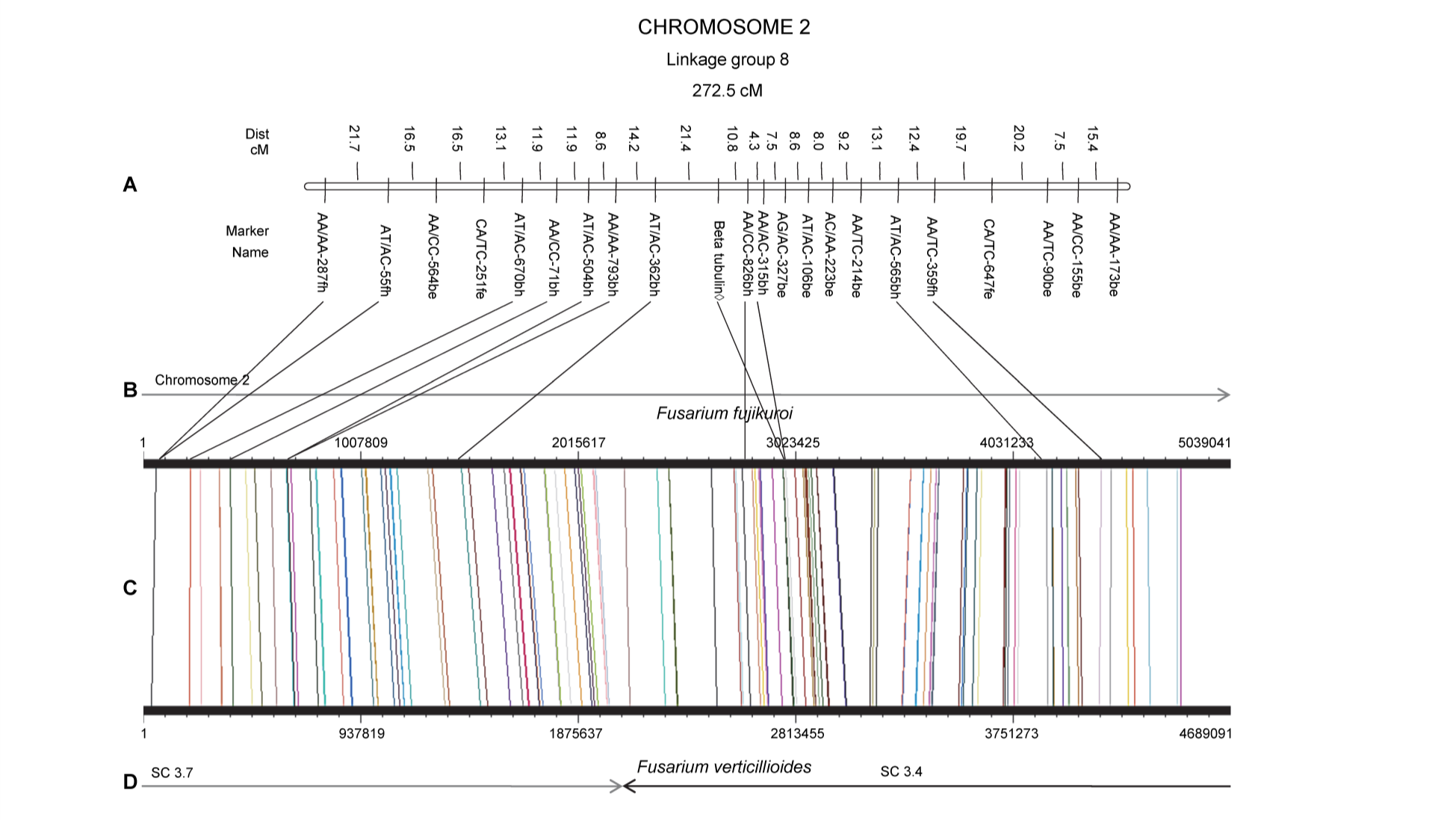


Figure S2.


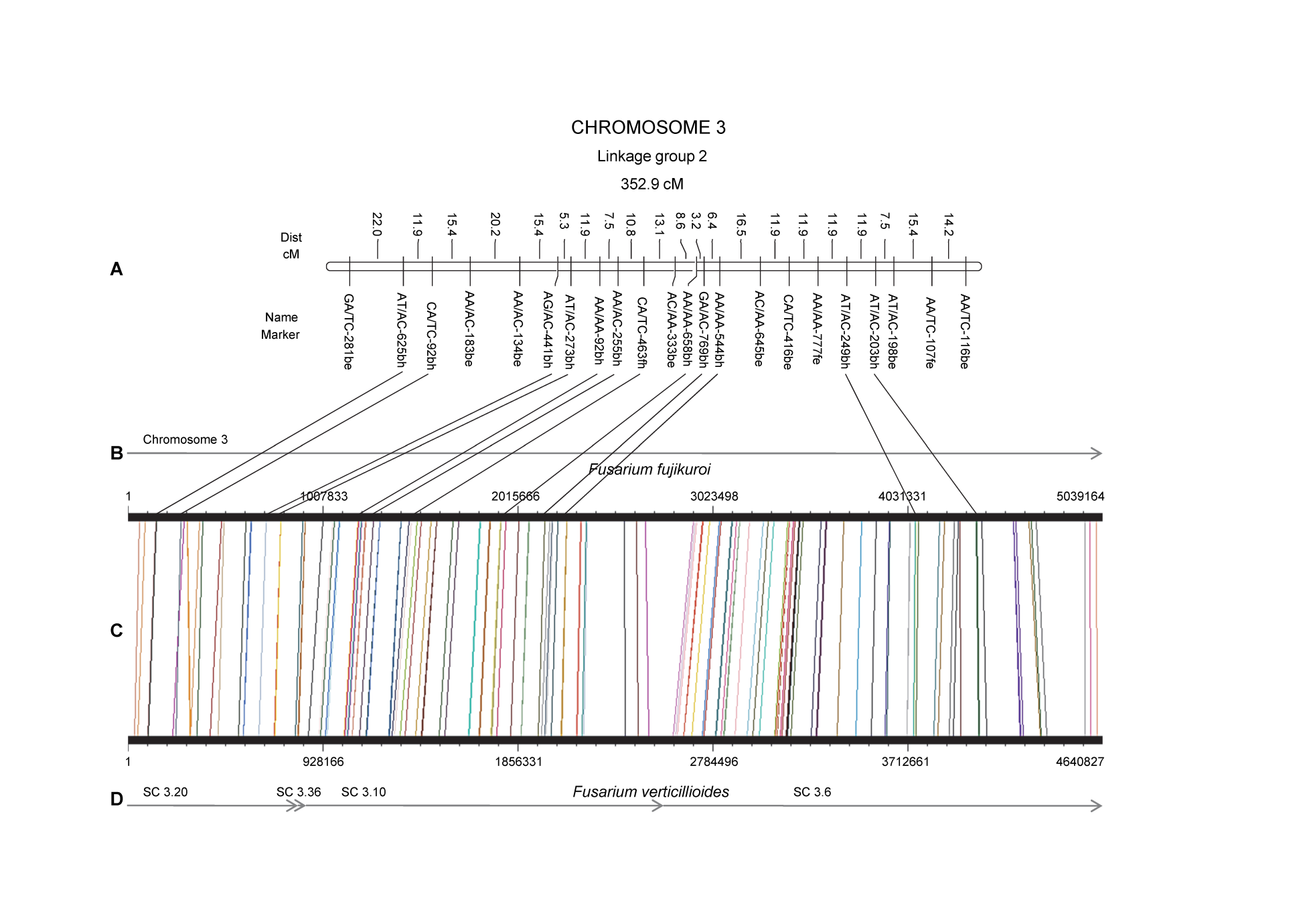


Figure S3.


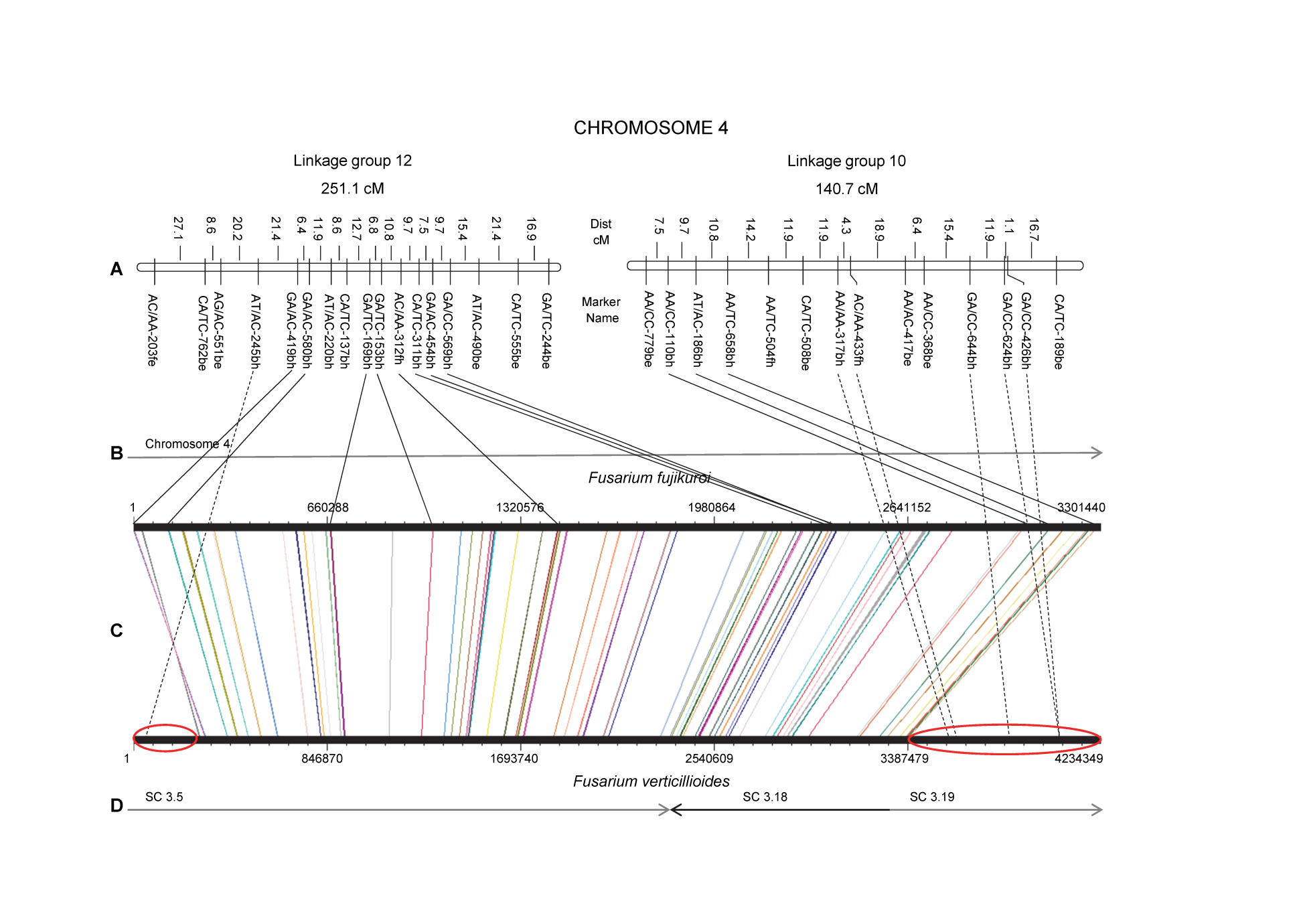


Figure S4.


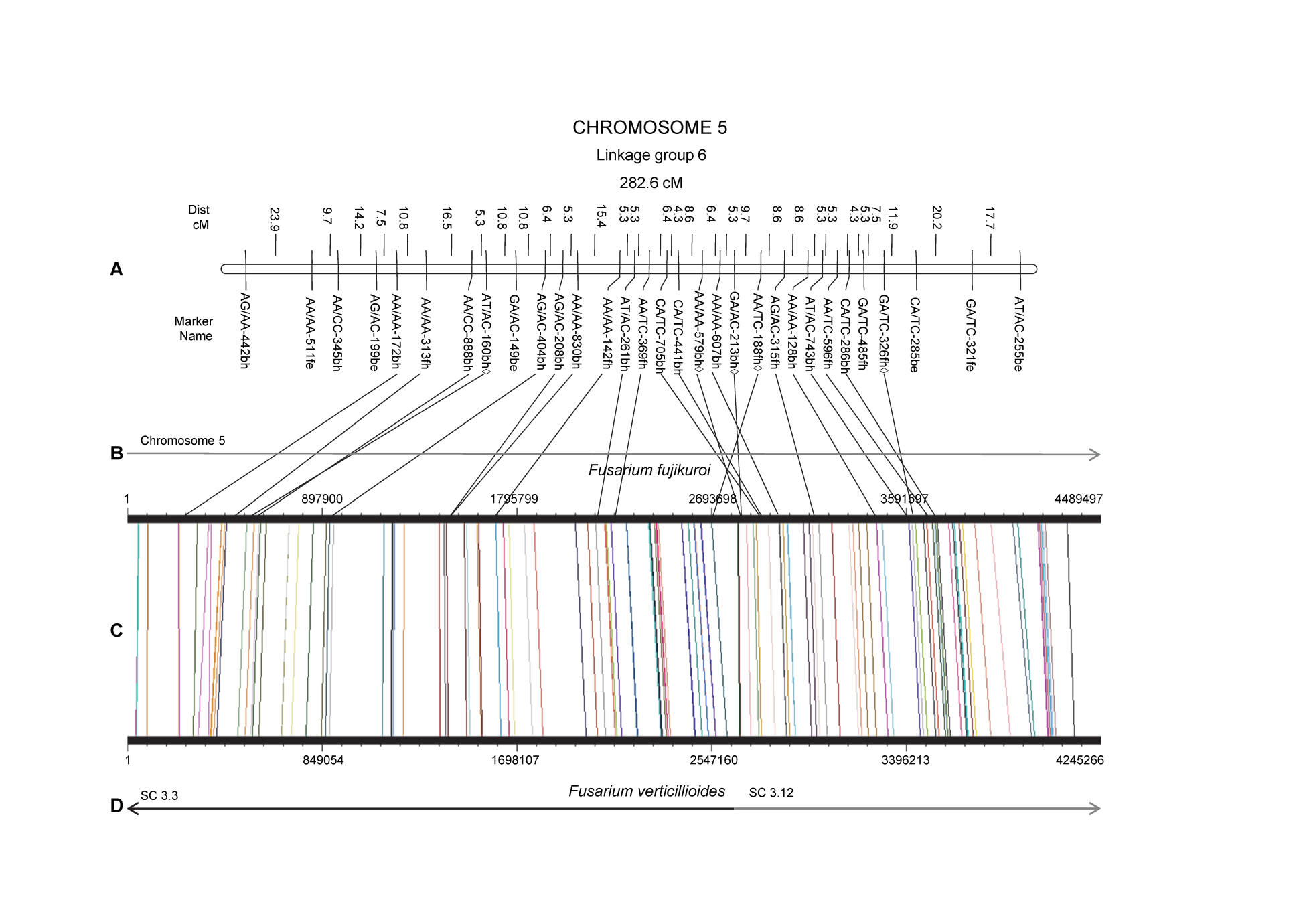


Figure S5.


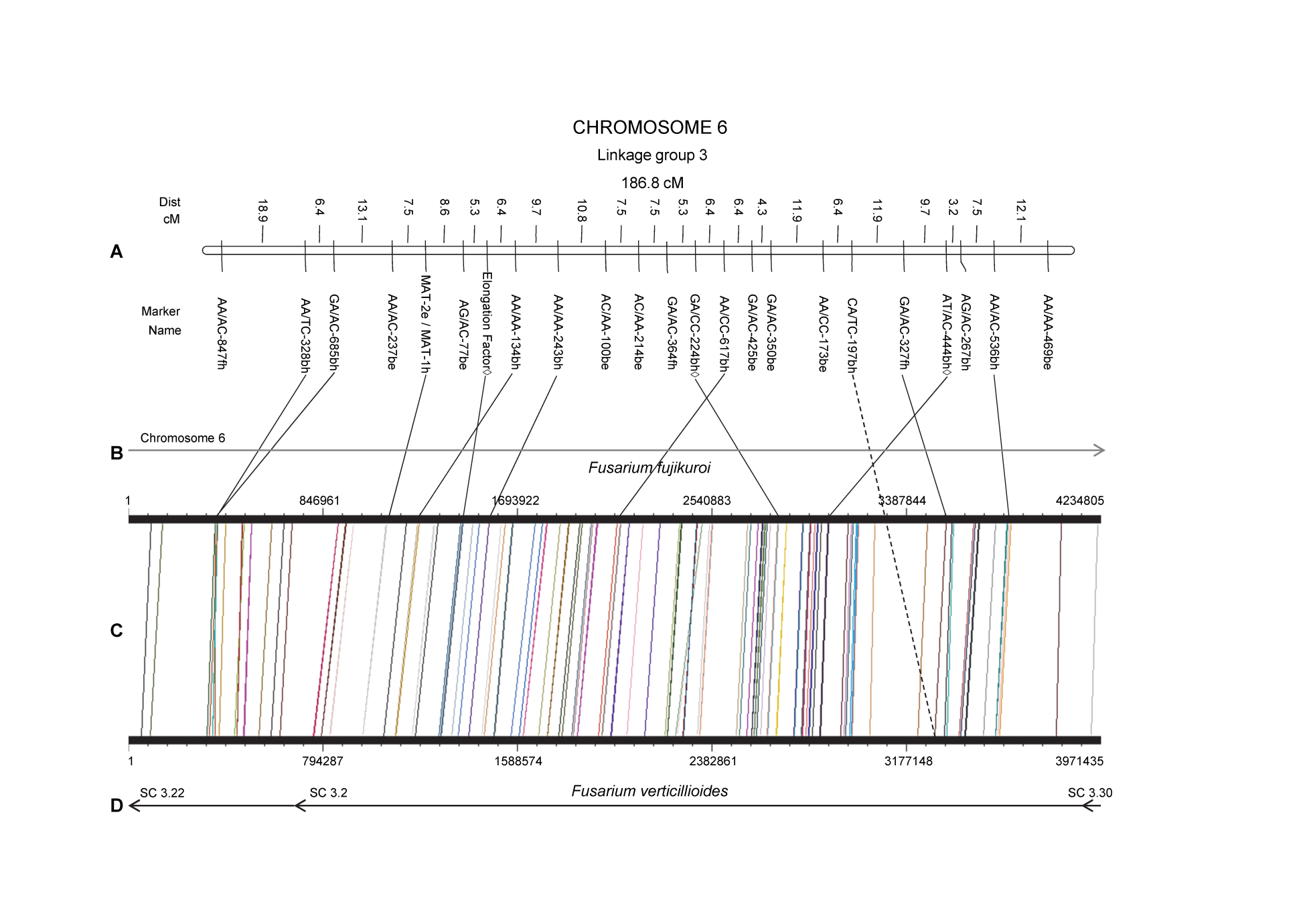


Figure S6.


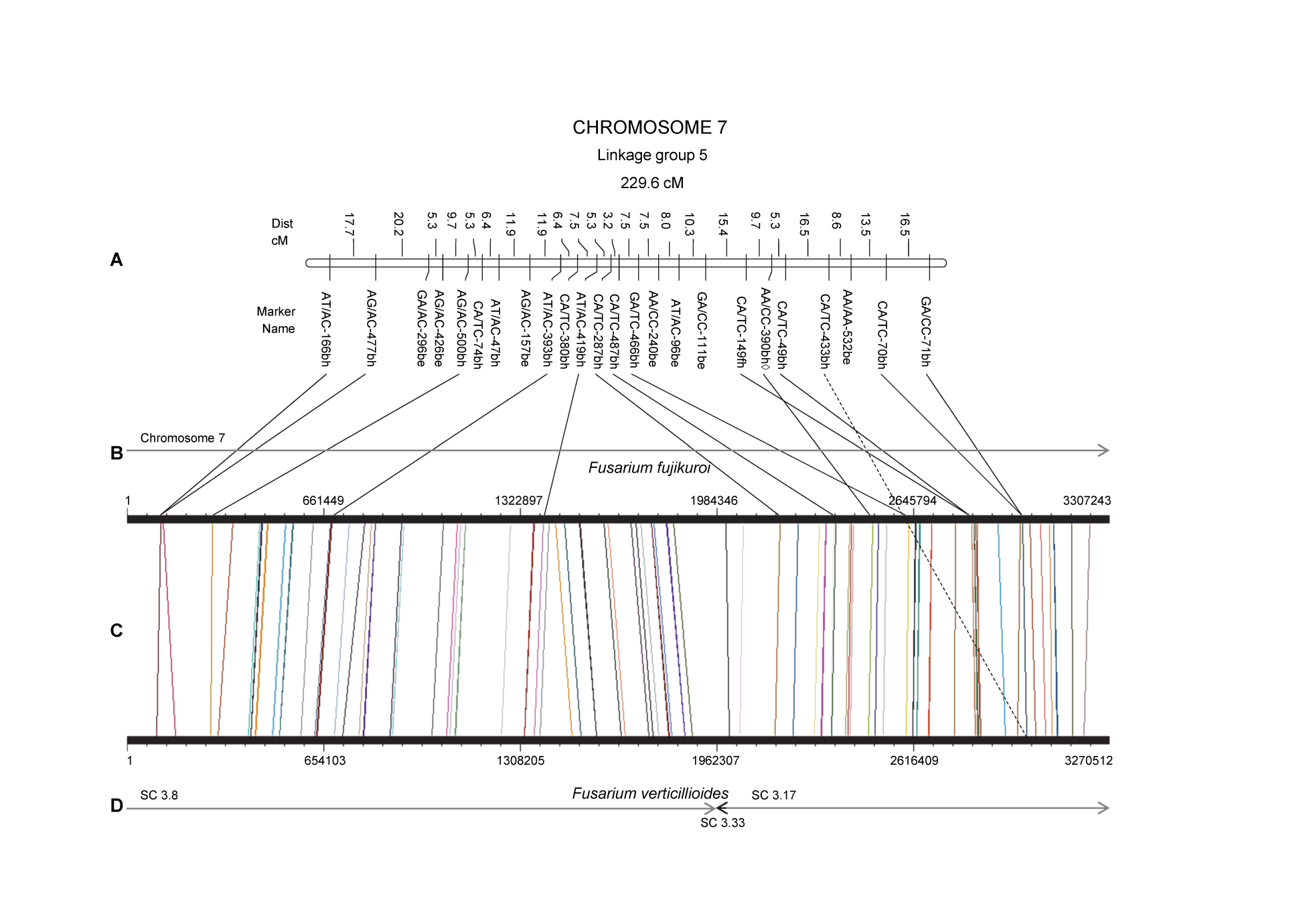


Figure S7.


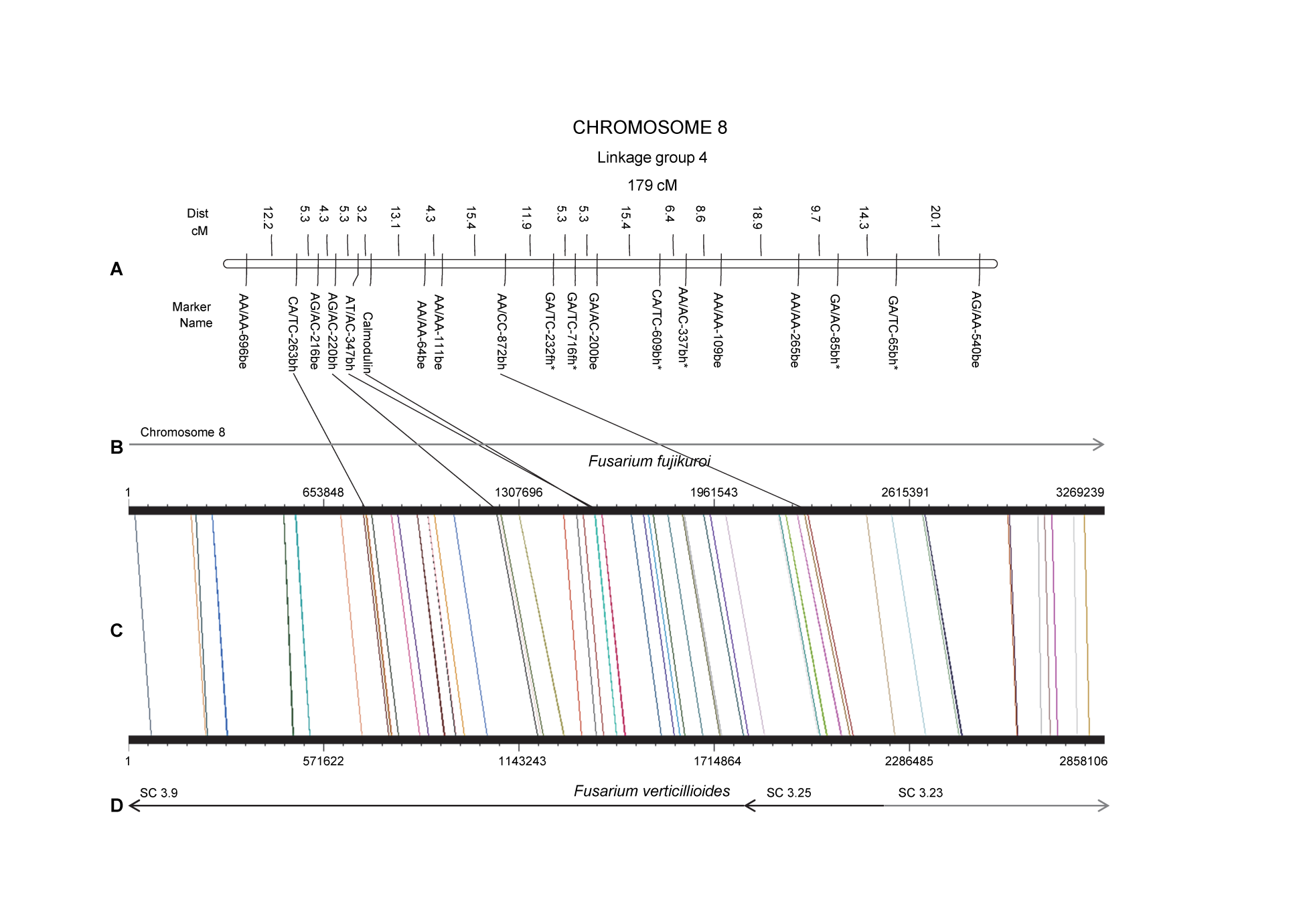


Figure S8.


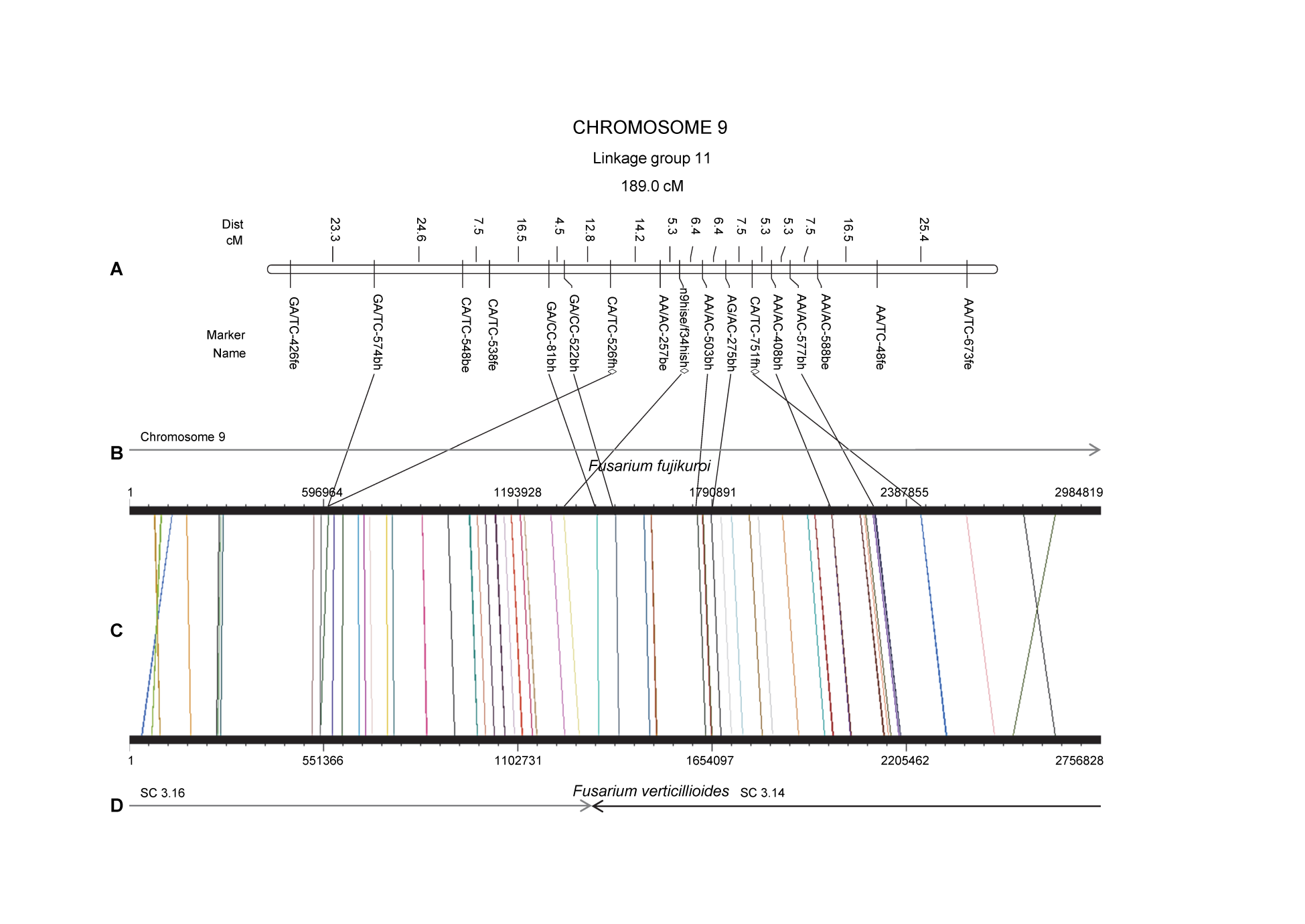


Figure S9.


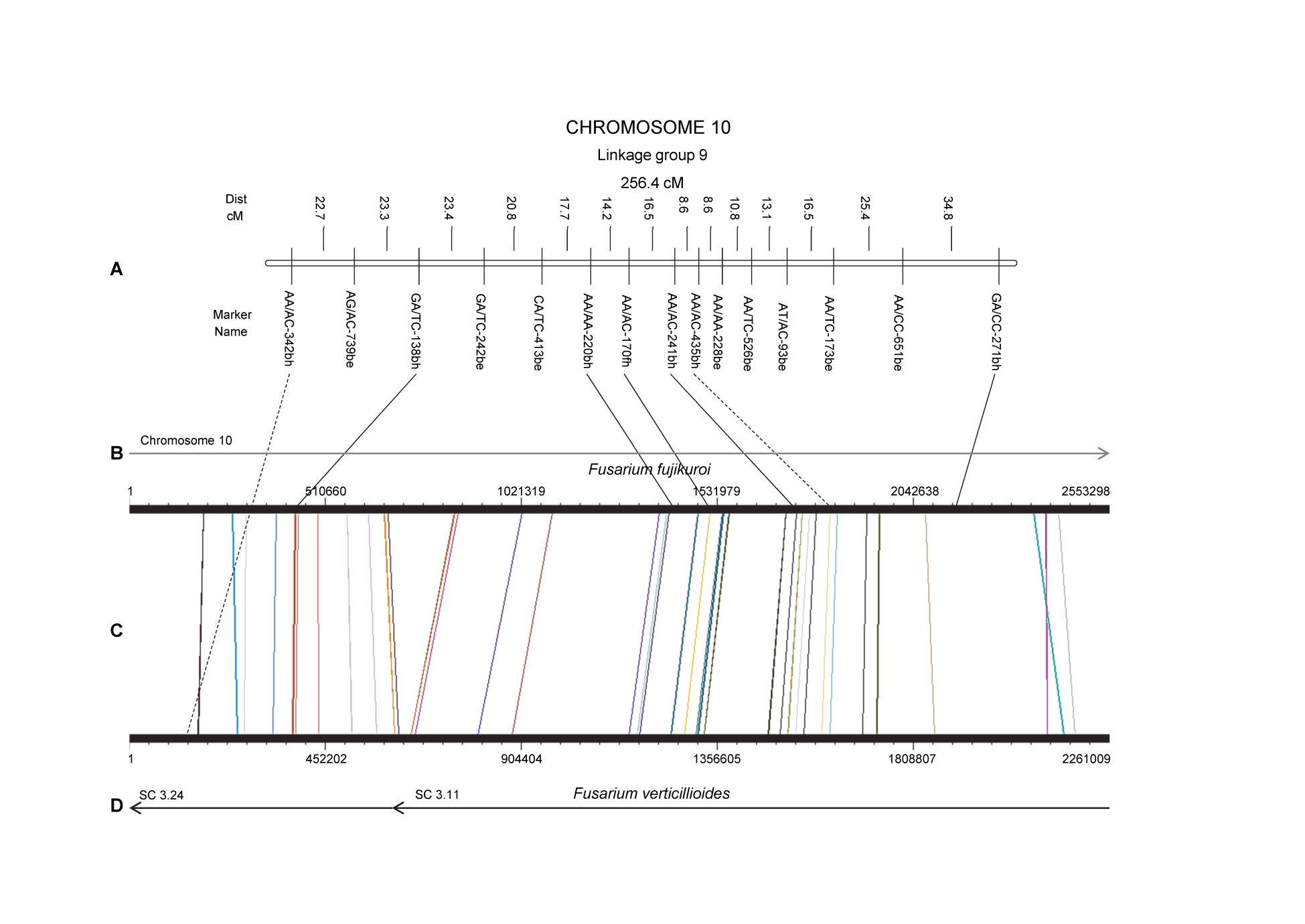

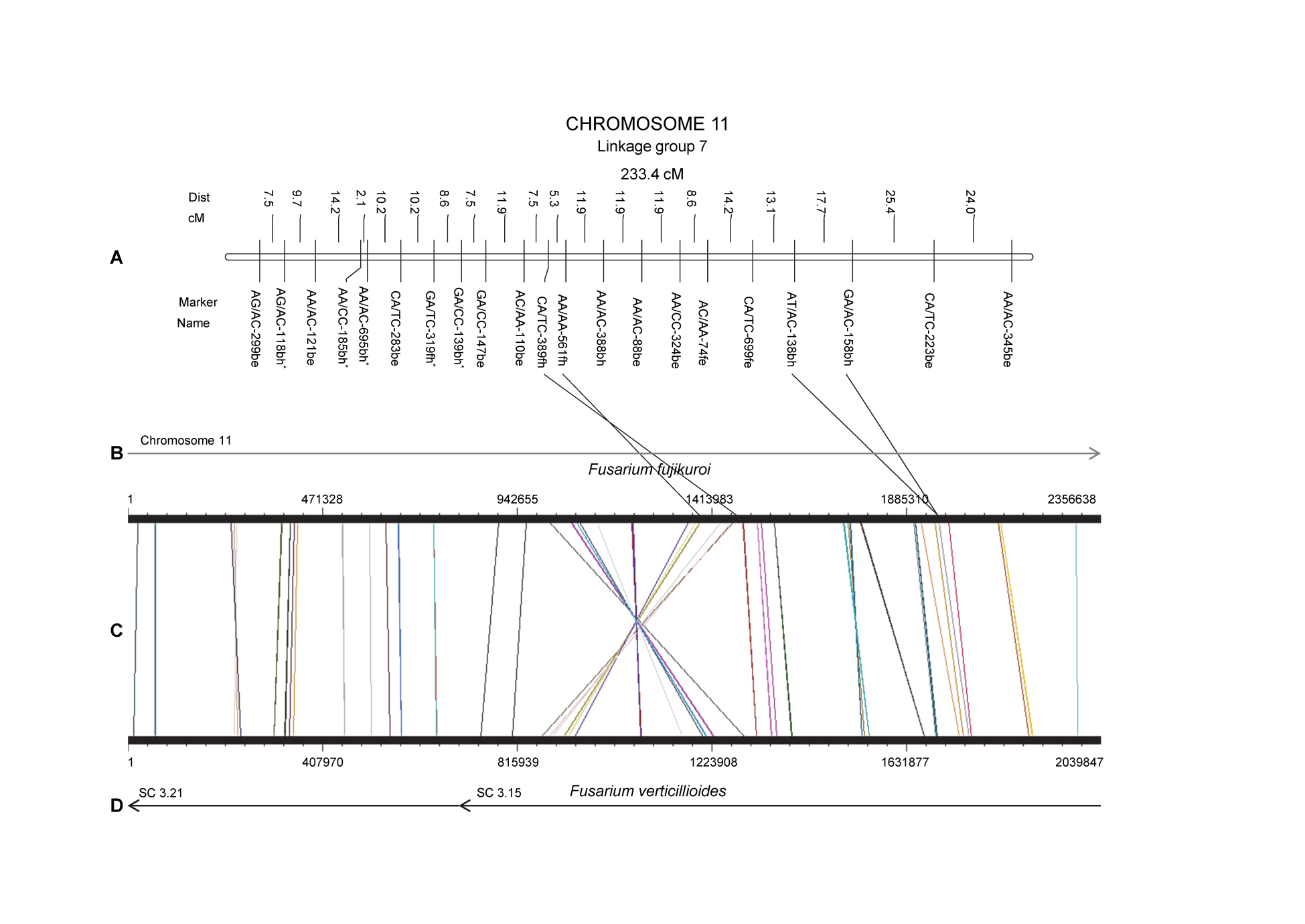


Figure S10.

Figure S11.
