# Supplementary material for: Genome-Wide Macrosynteny among Fusarium Species in the Gibberella fujikuroi Complex Revealed by Amplified Fragment Length Polymorphisms
Source: PLoS One. 2014 Dec 8;9(12):e114682. doi: 10.1371/journal.pone.0114682 (PMC4259476; doi:10.1371/journal.pone.0114682)
Supplement: S4 Text — The origin of the reciprocal translocation. (DOCX) [file pone.0114682.s004.docx]

**Supporting information file 4**

**The origin of the reciprocal translocation**


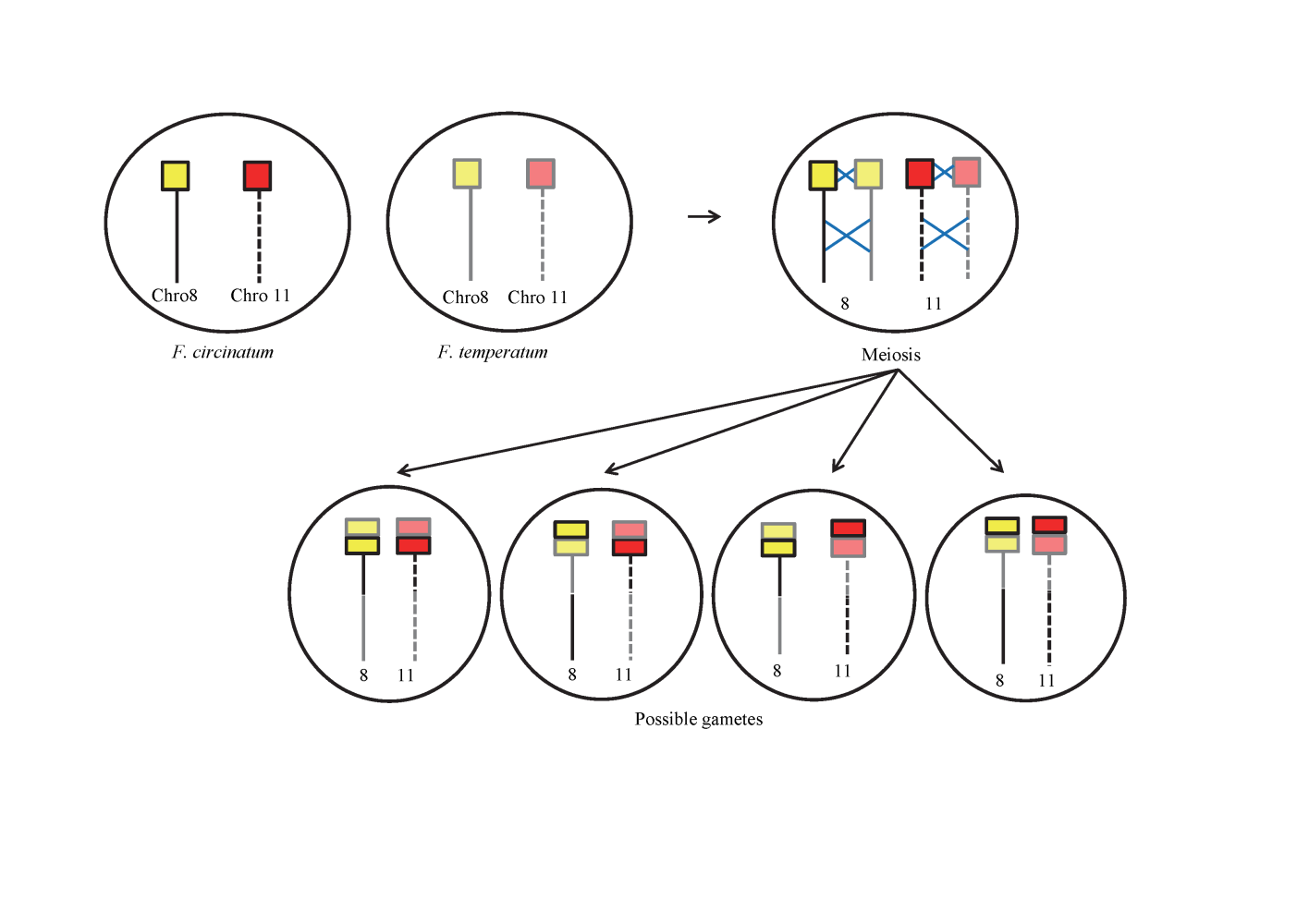


**Supplemental Figure 1.** Origin of the translocation. If the translocation is present in both parents, all progeny will have the translocation in both chromosome 8 and 11. During the production of each progeny, recombination in the identified translocated region would have been possible (but not required). Blocked areas indicate the translocation and blue crosses are representative of chiasmata. Black chromosomes represent the *F. circinatum* parent, whilst grey are representative of the *F. temperatum* parent. Chromosome 8 is indicated with a solid line and chromosome 11 with a dashed line.
