# Supplementary material for: Genome-Wide Macrosynteny among Fusarium Species in the Gibberella fujikuroi Complex Revealed by Amplified Fragment Length Polymorphisms
Source: PLoS One. 2014 Dec 8;9(12):e114682. doi: 10.1371/journal.pone.0114682 (PMC4259476; doi:10.1371/journal.pone.0114682)
Supplement: S5 Text — F. circinatum sequence identity to F. verticillioides and F. fujikuroi. (DOCX) [file pone.0114682.s005.docx]

**Supporting information file 5**

***F. circinatum* sequence identity to *F. verticillioides* and *F. fujikuroi***

Sequence identity was investigated using the program Geneious v7.0.4 (Biomatters, available from <http://www.geneious.com/>). Here, the LASTZ (Large-Scale Genome Alignment Tool) [1] plugin was used to identify homologous regions between the *F. circinatum* contigs containing pyrosequenced and/or *in silico* generated AFLP fragments, and the genomes of *F. verticillioides* and *F. fujikuoroi* (Supplemental Table 1). The total length of the identical regions were determined and expressed as a percentage of the total length of the *F. circinatum* contig under examination.

**Supplemental Table 1.** Summary of *Fusarium circinatum* contigs containing pyrosequenced and *in silico* generated AFLP fragments allocated to their respective chromosomes.

| Chromosome | Number of contigs^1^ | Total length (bp)^2^ | Average sequence identity (%) of *F. circinatum* contigs to other *Fusarium* scaffolds | |
| --- | --- | --- | --- | --- |
|  |  |  |  |  |
|  |  |  | *F. verticillioides*^3^ | *F. fujikuroi*^4^ |
| 1 | 113 | 2 943 579 | 88.76 | 88.75 |
| 2 | 77 | 1 893 799 | 88.64 | 89.69 |
| 3 | 82 | 2 317 096 | 87.84 | 87.96 |
| 4 | 70 | 2 021 401/  1 261 206^5^ | 85.83^***^ | 62.76^***^ |
| 5 | 80 | 1 982 418 | 88.25 | 88.24 |
| 6 | 75 | 1 813 417 | 87.29 | 87.47 |
| 7 | 58 | 1 579 082 | 87.58 | 87.77 |
| 8 | 46 | 1 375 216 | 88.53 | 88.41 |
| 9 | 44 | 1 005 555 | 87.14 | 87.19 |
| 10 | 33 | 900 045 | 82.78 | 83.78 |
| 11 | 40 | 1 826 093 | 82.16 | 81.78 |
|  |  |  |  |  |
| Total | 718 | 19 657 701 | (86.80^6^ | 85.43^6^) |

^1^Number of *F. circinatum* contigs that the pyrosequenced and *in silico* generated AFLP fragments were distributed over.

^2^Total length of the contigs from (1).

^3^Average sequence identity (%) between the *F. circinatum* contigs (1) and the *F. verticillioides* scaffolds.

^4^Average sequence identity (%) between *F. circinatum* contigs (1) and the *F. fujikuroi* scaffolds.

^5^*Fusarium fujikuroi* chromosome 4 is *ca.* 0.9 Mb smaller to the corresponding *F. verticillioides* chromosome [2].

^6^Average sequence identity over all the chromosomes.

^*^Significant deviation between sequence similarity of *F. circinatum* to *F. verticillioides*, and *F. circinatum* to *F. fujikuroi*. Significant deviation is noted as follows: ^*^ 5%, ^**^ 1% and ^***^ 0.1%.

**References**

1. Harris RS (2007) Improved pairwise alignment of genomic DNA. Pennsylvania, USA: Pennsylvania State University. 84 p.

2. Wiemann P, Sieber CMK, Von Bargen KW, Studt L, Niehaus E-M, et al. (2013) Unleashing the cryptic genome: Genome-wide analyses of the rice pathogen *Fusarium fujikuroi* reveal complex regulation of secondary metabolism and novel metabolites. PLoS Pathogens: e1003475.
